# Phenome-wide association study (PheWAS) of colorectal cancer risk SNP effects on health outcomes in UK Biobank

**DOI:** 10.1038/s41416-021-01655-9

**Published:** 2021-12-15

**Authors:** Xiaomeng Zhang, Xue Li, Yazhou He, Philip J. Law, Susan M. Farrington, Harry Campbell, Ian P. M. Tomlinson, Richard S. Houlston, Malcolm G. Dunlop, Maria Timofeeva, Evropi Theodoratou

**Affiliations:** 1 Centre for Global Health, Usher Institute, University of Edinburgh, Edinburgh, UK; 2 School of Public Health and the Second Affiliated Hospital, Zhejiang University, Hangzhou, China; 3 Colon Cancer Genetics Group, Cancer Research UK Edinburgh Centre and Medical Research Council Human Genetics Unit, Institute of Genetics and Cancer, University of Edinburgh, Edinburgh, UK; 4 Department of Oncology, West China School of Public Health and West China Fourth Hospital, Sichuan University, Chengdu, China; 5 Division of Genetics and Epidemiology, The Institute of Cancer Research, London, UK; 6 Cancer Research UK Edinburgh Centre, Institute of Genetics and Cancer, University of Edinburgh, Edinburgh, UK; 7 Danish Institute for Advanced Study (DIAS), Department of Public Health, University of Southern Denmark, Odense, Denmark

**Keywords:** Cancer epigenetics, Cancer epidemiology, Colorectal cancer

## Abstract

**Background:**

Associations between colorectal cancer (CRC) and other health outcomes have been reported, but these may be subject to biases, or due to limitations of observational studies.

**Methods:**

We set out to determine whether genetic predisposition to CRC is also associated with the risk of other phenotypes. Under the phenome-wide association study (PheWAS) and tree-structured phenotypic model (TreeWAS), we studied 334,385 unrelated White British individuals (excluding CRC patients) from the UK Biobank cohort. We generated a polygenic risk score (PRS) from CRC genome-wide association studies as a measure of CRC risk. We performed sensitivity analyses to test the robustness of the results and searched the Danish Disease Trajectory Browser (DTB) to replicate the observed associations.

**Results:**

Eight PheWAS phenotypes and 21 TreeWAS nodes were associated with CRC genetic predisposition by PheWAS and TreeWAS, respectively. The PheWAS detected associations were from neoplasms and digestive system disease group (e.g. benign neoplasm of colon, anal and rectal polyp and diverticular disease). The results from the TreeWAS corroborated the results from the PheWAS. These results were replicated in the observational data within the DTB.

**Conclusions:**

We show that benign colorectal neoplasms share genetic aetiology with CRC using PheWAS and TreeWAS methods. Additionally, CRC genetic predisposition is associated with diverticular disease.

## Introduction

Colorectal cancer (CRC) is the third most commonly diagnosed cancer and the second leading cause of cancer deaths globally [1]. Most CRC cases (about 70–90%) are developed from benign or pre-malignant colorectal neoplasms following the adenoma-carcinoma pathway [2]. Inflammatory bowel disease is among the diseases that are reported to be associated with a higher risk of CRC [3]. Different CRC screening strategies exist for patients with colorectal adenoma or polyps, or inflammatory bowel disease [4, 5]. Meanwhile, associations between CRC and health outcomes outside the digestive system have been observed in prospective observational studies, including metabolic syndrome [6], type 2 diabetes mellitus [7], chronic liver diseases [8], schizophrenia [9] and rheumatoid arthritis [10]. However, the direction and the magnitude of the associations are still unclear. Understanding the associations between CRC and other health outcomes could improve prevention, early detection and management of CRC as well as other health outcomes related to CRC.

Genome-wide association studies (GWASs) have identified over 100 susceptibility loci associated with CRC risk [11, 12]. These genetic variants combined into a polygenic risk score (PRS) can be used as a measure of genetic predisposition to CRC. By applying a phenome-wide association framework, we can explore genotype-phenotype associations using the CRC PRS as the risk factor. Therefore, in this study, we aim to explore phenotypes that are associated with CRC genetic predisposition under the phenome-wide association framework, leveraging the PRS for CRC risk.

## Methods

### Dataset

The UK Biobank (UKBB) is a prospective cohort study of around 500,000 volunteers resident in the UK, aged from 40 to 69, who were recruited between 2006 and 2010. A wide range of data has been collected on participants including genetic data, electronic medical records (cancer registry, death registry, hospital inpatient data and primary care data), biomarker measurements and other risk factors [13]. Genotyping, quality control and genotype imputation were conducted by the UKBB team before the data release and the procedure is described by Bycroft et al. [14]. Briefly, the initial 50,000 participants were genotyped by the Affymetrix UK BiLEVE Axiom array and the remaining 450,000 participants were genotyped by the Affymetrix UKBB Axiom array. Genotype imputation was performed using a merged reference panel of the Haplotype Reference Consortium (HRC) and the UK10K haplotype resources. For a total of 488,366 participants in the UKBB with genotype data, the current study is restricted to a subgroup of 339,256 genetically unrelated white British with high-quality genotype data. To minimise associations due to reverse causality, CRC cases were removed. A total of 334,385 individuals were included in the main analysis. More details on UKBB and the data quality control are given in supplementary methods.

### CRC polygenic risk score

Two recent large CRC GWAS studies (Huyghe et al. [11] and Law et al. [12]) were used to extract a total of 221 unique CRC risk associated SNPs. For duplicated SNPs, we kept the effect estimate for the variant with the smallest *P*-value. Both, newly detected variants and known variants from previously published GWASs summarised by Huyghe et al. and Law et al were used to generate PRS [11, 12]. A total of 127 SNPs were retained to generate the CRC PRS, after we excluded missing SNPs, ambiguous AT/CG variants and those in linkage disequilibrium (LD, R^2^ > 0.2) based on the 1000 genomes European reference panel (Fig. S1, Table S1). The PRS was created by adding the weighted (by the effect estimate of each SNP) dosages of risk alleles for each of the 127 SNPs (CRC PRS_127_). The estimated total variance in CRC risk explained by these 127 SNPs was 30.6% (supplementary methods). The SNP effect estimates were extracted from the GWAS of Huyghe et al. [11], excluding UKBB samples. We also re-ran our previous meta-analysis of 15 CRC GWASs but excluded UKBB data to generate effect estimates for SNPs extracted from Law et al. [12]. The correlations between the CRC PRS_127_ and CRC risk were then tested using UKBB data (which includes 4871 CRC cases at 31/03/2017).

### Phenome-wide association framework

**Fig. 1:**
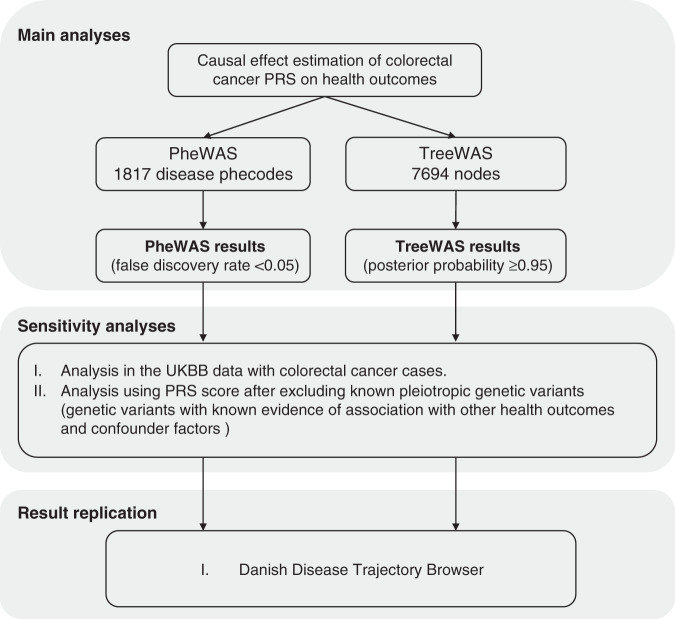
Schematic representation of the study design. PheWAS phenome-wide association study, TreeWAS tree-structured phenotypic model, PRS polygenic risk score, UKBB UK Biobank.

The rationale of the study design is presented in Fig. 1. We used records from the cancer registry, death registry and hospital inpatient statistics. The details of phenotyping were presented in supplementary methods. All cases in the three datasets were classified according to the International Classification of Disease (ICD) version 9 and 10. All phenotypes categorised into ‘Symptoms, signs and abnormal clinical and laboratory findings, not elsewhere classified’ (ICD10, chapter XVIII), ‘Injuries and poisonings and certain other consequences of external causes’ (ICD10, chapter XIX), ‘External causes of morbidity and mortality’ (ICD10, chapter XX), ‘Factors influencing health status and contacts with health services’ (ICD10, chapter XXI) and ‘Codes for special purposes’ (ICD10, chapter XXII) groups were removed from the analyses.

We combined the records of all three datasets and translated the ICD codes into PheCODE groups using previously described classification, which included 1817 hierarchical PheCODEs categorised into 17 components [15, 16]. The PheCODE system combines correlated ICD codes into a distinct code and automatically excludes patients with related diseases from the corresponding control groups [16]. We performed multivariable logistic regression analysis, adjusting for age, sex, assessment centre and the first 10 genetic principal components. We conducted a power estimation for the PheWAS analysis [17]. We corrected the *P*-values for multiple testing using the false discovery rate (FDR) with an FDR q-value threshold of 0.05 [18]. Subsequently, for all significant associations, we estimated the odds ratio (OR) of the case odds between the top and bottom quartiles of CRC PRS_127_ and tested the null hypothesis of no differences between the quartiles using a chi-square test [19]. The PheWAS analysis on CRC predisposition polymorphisms and multiple diseases was performed using the PheWAS R package (R version 3.6.1) [16].

Next, we re-analysed the data using TreeWAS [20], which is an approach to estimate the associations of genetic variants with disease phenotypes by reducing the dimension and heterogeneity of the outcome data. Compared to PheWAS, the TreeWAS method considers the genetic correlations across phenotypes in a Markov process, and therefore, has more power to detect associations (about 20% higher power) [20]. Unlike the PheWAS, the TreeWAS analysis uses the ICD codes directly. In the TreeWAS analysis, associations between CRC predisposition polymorphisms and each node (terminal and internal nodes) of the disease tree structure were examined using a Bayesian analysis framework. A Bayes factor statistic (BF_tree_) was estimated to indicate non-zero for at least one node and a marginal posterior probability (PP) was estimated for each node to indicate non-zero using a maximum posteriori estimator. The thresholds were set at PP ≥ 0.95 and log_10_(BF_tree_) > 1. Finally, for all the significant associations, we estimated the OR of the case odds between the top and bottom quartiles of CRC PRS_127_ and tested the null hypothesis using a chi-square test. The analysis was performed using the R script based on R environment version 3.6.1 [20].

### Sensitivity analysis

We performed two sensitivity analyses. First, we identified SNPs with possible pleiotropic effects with a threshold at *P* < 1 × 10^−5^ through searching both the National Human Genome Research Institute (NHGRI)-European Bioinformatics Institute (EBI) Catalog [21] and PhenoScanner [22, 23] for published GWASs (accessed on 27 July 2021). Then, we created a CRC-specific PRS with 53 SNPs after excluding 74 SNPs with potential pleiotropic effects. We repeated the PheWAS and TreeWAS analyses using the CRC-specific PRS (CRC PRS_53_). Second, we re-ran the PheWAS and TreeWAS analysis by including CRC cases in the dataset.

### Results replication in Danish Disease Trajectory Browser

The Danish Disease Trajectory Browser (DTB) is a tool that allows the identification of statistically significant associations among diseases coded by ICD10 in a dataset of 7.2 million patients and 122 million admissions from the Danish National Patient Register and Danish Register for Causes of Death [24]. DTB can also create disease trajectories reflecting sequential disease progression patterns in a data-driven manner. The disease progression patterns were confirmed when the direction of diagnoses was statistically significant compared to the reverse direction. The minimum number of cases for each disease was restricted to 20. More details on DTB can be found in the published paper [24] and the supplementary methods. To validate the observed associations between genetic predisposition to CRC and other diseases, we searched the DTB using ICD10 codes for CRC (C18, C19 and C20) to uncover potential causal, co-occurring or interacting associations for CRC in this large-scale, population health cohort.

For any discrepancy in the direction of significant associations between DTB and our analysis, we performed genetic correlation and bi-directional Mendelian randomisation (MR) analyses to further test the direction of the association. The correlation analysis was performed by using the ‘cor.test’ function of R. The causal effects and the corresponding standard errors of exposures on outcomes were calculated by using the random effects inverse variance-weighted method [25]. MR-Egger was applied to explore any potential bias introduced by pleiotropy [26]. Further details of the MR analyses are presented in supplementary methods.

We investigated whether significant associations identified through the sensitivity analysis of including CRC cases and replicated in DTB, were due to co-occurrence with CRC. First, we stratified our dataset into ten equal groups by CRC PRS_127_ deciles and calculated the proportion of cases in each decile for each phenotype. Subsequently, we compared the difference of the case proportions in the highest and lowest CRC PRS_127_ decile between the datasets with and without CRC cases by performing paired *t*-tests. When the tested results were not significant, the observed associations were likely due to co-occurrence. The threshold of *P*-value was set at 0.05. All analyses were performed in R (version 3.6.1).

## Results

A total of 339,256 unrelated White British UKBB participants were retained after sample quality control and 334,385 after removing CRC cases (Fig. S2). The mean age of the study population was 56.8 (standard deviation [SD]: 8.0); mean BMI was 27.4 (SD: 4.8) kg/m^2^; 46.2% of participants were male (Table S2). The association between CRC PRS_127_ and CRC status in UKBB is presented in Table S3.

**Fig. 2:**
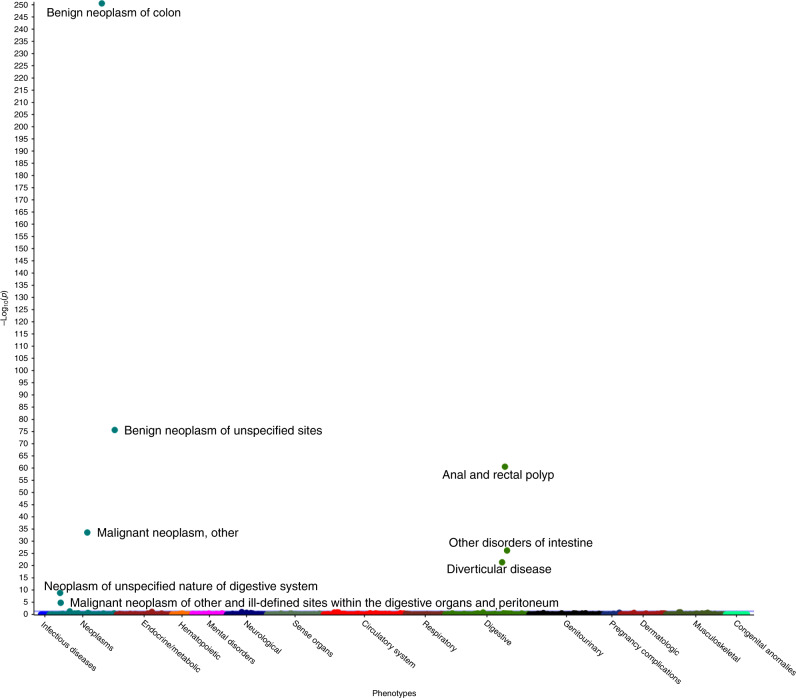
PheWAS results of the associations between weighted polygenic risk scores of colorectal cancer and other diseases in the UK Biobank. The purple line indicates the threshold of statistical significance (false discovery rate q < 0.05).

A total of 11,544 unique ICD10 and 3109 ICD9 codes were summarised from hospital inpatient, cancer registry, and death registry data of the UKBB cohort. These codes were mapped to 1647 distinct PheCODEs after excluding diseases categorised as ‘injuries & poisonings’ and ‘symptom’. We restricted the analysis to PheCODEs with at least 20 cases [15, 27]. An estimated total of 679 cases per outcome was needed to have 80% power to detect an OR of 1.20; and 20 cases per outcome to detect an OR of 2.15. Finally, associations between CRC predisposition polymorphisms and 1326 PheCODEs grouped into 15 disease categories (median number of cases: 385 [range: 20–119,971], Table S4) were analysed. About 38.75% of PheCODEs had more than 679 cases. Eight PheCODEs were associated with CRC PRS_127_ at FDR q < 0.05 (Table 1, Fig. 2). These PheCODEs belonged to colorectal neoplasms and digestive system disease groups, such as benign neoplasm of colon (FDR q = 3.94 × 10^−251^), anal and rectal polyp (FDR q = 2.43 × 10^−61^) and diverticular disease (FDR q = 4.02 × 10^−22^) (Fig. 2). A high CRC PRS was associated with an increased risk of having benign neoplasm of colon (OR_top vs bottom PRS quartiles_: 1.93, 95% CI: 1.85, 2.01), anal and rectal polyp (OR_top vs bottom PRS quartiles_: 1.66, 95% CI: 1.54, 1.78) and diverticular disease (OR_top vs bottom PRS quartiles_: 1.18, 95% CI: 1.14, 1.22) (Table 1).

**Table 1:** Results of PheWAS and effect estimates of the comparison between the top risk quartile and the bottom risk quartile.

Description	Correspond ICD10 codes	Group	Number of participants	Number of cases	Beta	SE	FDR q	OR (95% CI)^a^	*P*^a^
Benign neoplasm of colon	D12, K63.5	Neoplasms	334,134	18,796	0.46	0.01	3.94 × 10^−251^	1.93 (1.85, 2.01)	3.59 × 10^−201^
Benign neoplasm of unspecified sites	D10–D36	Neoplasms	334,385	40,252	0.18	0.01	2.09 × 10^−76^	1.29 (1.25, 1.33)	1.05 × 10^−63^
Anal and rectal polyp	K62.0, K62.1	Digestive	268,006	6807	0.37	0.02	2.43 × 10^−61^	1.66 (1.54, 1.78)	9.06 × 10^−47^
Malignant neoplasm, other^b^	B21.7, C00–C97, D00–D48, M90.7	Neoplasms	328,419	91,309	0.09	0.01	2.60 × 10^−34^	1.13 (1.11, 1.16)	1.67 × 10^−29^
Other disorders of intestine	K00–K14, K55–K63, K92.8, K92.9	Digestive	332,441	71,242	0.09	0.01	6.12 × 10^−21^	1.13 (1.10, 1.16)	5.36 × 10^−25^
Diverticular disease	K57	Digestive	298,598	27,266	0.12	0.01	4.02 × 10^−22^	1.18 (1.14, 1.22)	2.48 × 10^−20^
Neoplasm of unspecified nature of digestive system	D37	Neoplasms	323,276	953	0.39	0.06	2.19 × 10^−9^	1.91 (1.59, 2.30)	3.25 × 10^−12^
Malignant neoplasm of other and ill-defined sites within the digestive organs and peritoneum	C26.1, C26.8, C26.9, D01, D01.7, D01.9	Neoplasms	324,092	1769	0.23	0.04	1.71 × 10^−5^	1.31 (1.15, 1.49)	4.50 × 10^−5^

*SE* standard error, *FDR* false discovery rate, *OR* odds ratio, *CI* confidence interval.

^a^The comparison of case frequency between the top risk quartile and the bottom risk quartile.

^b^Colorectal cancer cases were removed from this PheCODE.

**Fig. 3:**
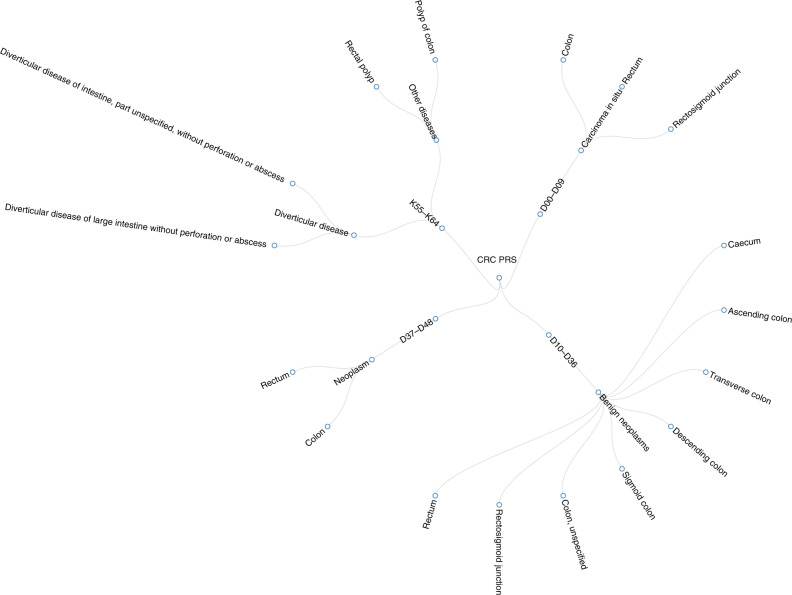
TreeWAS results of the associations between weighted polygenic risk scores of colorectal cancer and other diseases in UK Biobank. The blue circles represent the disease nodes associated with colorectal cancer polygenic risk score (CRC PRS). D00–D09: in situ neoplasms, D10–D36: benign neoplasms, D37–D48: neoplasms of uncertain or unknown behaviour, K55–K64: other diseases of intestines.

TreeWAS analysis identified 21 nodes in four disease blocks that had a PP ≥ 0.95 based on ICD10 diagnosed terms (Table 2, Fig. 3). The TreeWAS results were consistent with the PheWAS results and had the same direction of effect. The significant associations were limited to neoplasms and diseases of the digestive system (Table S4), such as in situ neoplasms of colon (PP = 1.00), benign neoplasms of colorectum (PP = 1.00), diverticular disease (PP = 1.00), rectal polyps (PP = 1.00) and colon polyps (PP = 1.00). Compared to PheWAS results, the TreeWAS analysis detected more subgroup associations (Tables 1 and 2). When comparing the case frequency between the top and bottom risk quartiles, we found the effect of CRC PRS_127_ on the outcome “colon polyps” was the strongest (P = 2.09 × 10^−104^). The outcome “rectal polyps” had a stronger OR_top vs bottom PRS quartiles_ (95% CI) compared to combined anal and rectal polyps, 1.79 (1.66, 1.93) with *P*-value of 6.37 × 10^−53^ versus 1.66 (1.54, 1.78) with *P*-value of 9.06 × 10^−47^.

**Table 2:** Results of TreeWAS and effect estimates of the comparison between the top risk quartile and the bottom risk quartile.

	TreeWAS analysis				Comparison of odds of disease between the top CRC risk quartile and the bottom risk quartile	
Meaning	max_b	b_ci_lhs	b_ci_rhs	POST_ACTIVE	OR (95% CI)	*P*
Block D00–D09 in situ neoplasms	0.03	0.03	0.05	0.86	/	/
D01 Carcinoma in situ of other and unspecified digestive organs	0.95	0.70	1.20	1.00	/	/
D01.0 Colon	0.95	0.70	1.20	1.00	4.67 (2.27, 9.59)	7.41 × 10^−06^
D01.1 Rectosigmoid junction^a^	0.95	0.70	1.20	0.96	/	/
D01.2 Rectum	0.95	0.70	1.20	1.00	2.83 (1.47, 5.47)	1.96 × 10^−03^
Block D10–D36 Benign neoplasms	0.03	0.03	0.05	0.90	/	/
D12 Benign neoplasm of colon, rectum, anus and anal canal	0.63	0.61	0.66	1.00	/	/
D12.0 Caecum	0.63	0.61	0.66	1.00	2.59 (2.24, 3.00)	3.64 × 10^−40^
D12.2 Ascending colon	0.63	0.61	0.66	1.00	2.82 (2.44, 3.25)	5.34 × 10^−50^
D12.3 Transverse colon	0.63	0.61	0.66	1.00	2.76 (2.44, 3.13)	6.51 × 10^−62^
D12.4 Descending colon	0.63	0.61	0.66	1.00	2.73 (2.31, 3.23)	1.77 × 10^−34^
D12.5 Sigmoid colon	0.63	0.61	0.66	1.00	2.32 (2.12, 2.53)	1.09 × 10^−82^
D12.6 Colon, unspecified	0.63	0.61	0.66	1.00	2.16 (1.88, 2.47)	6.08 × 10^−30^
D12.7 Rectosigmoid junction	0.63	0.61	0.66	1.00	2.49 (1.78, 3.50)	7.02 × 10^−08^
D12.8 Rectum	0.63	0.61	0.66	1.00	2.60 (2.32, 2.92)	1.32 × 10^−63^
Block D37–D48 Neoplasms of uncertain or unknown behaviour	0.03	0.03	0.05	0.66	/	/
D37 Neoplasm of uncertain or unknown behaviour of oral cavity and digestive organs	0.03	0.02	0.99	0.85	/	/
D37.4 Colon	0.78	0.55	0.99	1.00	2.87 (1.91, 4.32)	1.94 × 10^−07^
D37.5 Rectum	0.80	0.60	1.16	1.00	4.23 (2.66, 6.73)	6.58 × 10^−11^
Block K55–K64 Other diseases of intestines	0.03	0.02	0.13	0.98	/	/
K57 Diverticular disease of intestine	0.11	0.08	0.14	1.00	/	/
K57.3 Diverticular disease of large intestine without perforation or abscess	0.11	0.08	0.14	1.00	1.19 (1.14, 1.23)	2.05 × 10^−19^
K57.9 Diverticular disease of intestine, part unspecified, without perforation or abscess	0.11	0.08	0.14	1.00	1.14 (1.06, 1.22)	2.90 × 10^−04^
K62 Other diseases of anus and rectum	0.03	0.02	0.12	0.63	/	/
K62.1 Rectal polyp	0.41	0.37	0.46	1.00	1.79 (1.66, 1.93)	6.37 × 10^−53^
K63 Other diseases of intestine	0.03	0.03	0.13	0.95	/	/
K63.5 Polyp of colon	0.43	0.40	0.46	1.00	1.84 (1.74, 1.95)	2.09 × 10^−104^

*max_b* maximum a posteriori effect estimates (beta) and the 95% credible interval (b_ci_lhs, b_ci_rhs), *POST_ACTIVE* posterior probability for the beta estimate in the tree analysis not being zero, *OR* odds ratio, *CI* confidence interval, *CRC* colorectal cancer.

^a^Number of cases in either the top risk quartile or the bottom risk quartile is 0.

The results using the CRC PRS_53_ (Table S5 and S6) were similar to the main analysis in both PheWAS and TreeWAS, but the effect estimates and *P*-values were attenuated. By including CRC cases in our dataset, strong associations between the CRC PRS_127_ and colon cancer (FDR q = 4.52 × 10^−167^), and CRC (FDR q = 5.08 × 10^−13^) were observed (Table S7 and S8). In addition, including CRC cases in the dataset introduced more associations with diseases outside the digestive system (e.g. diabetes mellitus, anaemia, renal failure, bacterial infection, gonarthrosis and secondary malignancies in lymph nodes, lungs, peritoneum or liver; Tables S7 and S8).

A total of 1274 disease trajectories were identified in DTB using ICD10 codes for CRC, among which 54 diseases were suggested to occur before CRC and 84 diseases were suggested to occur after a diagnosis of CRC (Table S9). All of the phenotypes identified in the DTB were covered by the UK Biobank dataset. Two out of the 54 precursors of CRC (i.e. benign neoplasm of colon and diverticular disease) and five out of the 84 diseases that were suggested to occur after CRC in DTB (i.e. benign neoplasm of colon, malignant neoplasm of the digestive organs, peritoneum and other and other diseases of intestine) were found to be associated with CRC PRS_127_ in our analysis.

The DTB suggested diverticular disease happens before CRC and we reported a significant association between CRC predisposition polymorphisms and diverticular disease. To further confirm the direction of the association between diverticular disease and CRC, we performed a genetic correlation analysis and a bi-directional MR analysis. The genetic variants for the diverticular disease were extracted from two GWASs [28, 29], and their effects on CRC were extracted from Law et al after removing UK Biobank (Table S10) [12]. We took the 127 genetic variants for CRC as the instrument of CRC and their effects on the diverticular disease were extracted from Schafmayer et al [28]. We found no significant correlation (*P* = 0.33) between CRC predisposition polymorphisms and diverticular disease predisposition polymorphisms with a correlation coefficient of 0.07 (Table S11). From the bi-direction MR, we found CRC to be causally associated with diverticular disease (OR [95% CI]: 1.008 [1.006, 1.010], *P* = 9.78 × 10^−18^) while no association at the opposite direction was observed (OR [95% CI]: 0.37 [0.10, 1.36], *P* = 0.14, Table S11).

Some of the associations between CRC PRS_127_ and non-digestive system diseases detected in the sensitivity analysis after including CRC cases were also observed in DTB (i.e. type 2 diabetes, anaemia, renal failure, bacterial infection, gonarthrosis and secondary malignancies in lymph nodes, lungs, peritoneum or liver). To test whether these associations were due to co-occurrence with CRC, we further described the case distribution in 10 CRC PRS_127_ deciles and compared the difference in proportions in the lowest versus the highest PRS risk decile between analyses with or without CRC cases for those phenotypes. We did not find a statistically significant difference for any of them (Fig. S3). For comparison, we also described the case distribution in PRS risk deciles for phenotypes detected by the main analysis (Fig. S3).

## Discussion

This study aimed to identify phenotypes that were associated with the genetic predisposition to CRC in the UKBB cohort under a phenome-wide association framework (i.e. PheWAS and TreeWAS). We conducted the main analysis in a dataset without CRC cases to minimise the possibility of identifying associations primarily caused by the presence of CRC cases or reverse causality and searched DTB to observe the association under an observational setting. In addition, we re-ran all analyses by including CRC cases in our dataset to avoid missing findings of unknown associations within CRC cases and we re-ran the analyses by using the CRC PRS_53_, which excluded pleiotropic SNPs, to check the robustness of our findings.

Not surprisingly, we found that the increased CRC PRS was associated with an increased risk of benign or pre-malignant colorectal neoplasms, and neoplasms of unspecified sites, suggesting a shared genetic background between CRC and pre-malignant colorectal neoplasms. These associations were detected by both PRSs (CRC PRS_127_ and CRC PRS_53_). These results suggested the potential benefits of polypectomy on CRC risk. A prospective study with 712 post colonoscopy CRC diagnoses during a 10-year follow-up time showed an inverse association between adenoma detection rate and post colonoscopy CRC risk [30]. Another two prospective studies reported similar findings [31, 32]. The US multi-Society Task Force on CRC has recommended endoscopic removal of colorectal lesions [33]. Our findings supported this recommendation. Additionally, screening of people with a family history of pre-malignant colorectal neoplasms may help to decrease the risk of CRC in those individuals [34, 35].

We found that an increased CRC PRS was associated with an increased risk of diverticular disease, whereas the DTB suggested diverticular disease happens before CRC. The differences in the clinical practice between the countries [36], the effect of cancer screening on the identification of diverticular disease were reported [37], and time of the process from genetic predisposition to disease occurrence, which may explain the observed discrepancy with the findings from the DTB. Our follow-up bi-directional MR analysis indicated a causal association between CRC and diverticular disease but not the reverse, which suggested shared aetiology for the two diseases and the importance of diverticular disease prevention and/or treatment in CRC patients and/or individuals with a higher risk of CRC. A meta-analysis including 11 cross-sectional studies, one case-control study and 2 cohort studies did not report a significant association between diverticular disease and CRC [38], which was consistent with our finding from the MR analysis. Meanwhile, the non-correlation between CRC genetic variants and diverticular disease genetic variants detected by our genetic correlation analysis and the stable effect estimates in the datasets with and without removing CRC cases suggested that the observed CRC-diverticular disease association was unlikely due to co-detection. However, it is noteworthy that diverticular disease shares risk factors with CRC, which may be a potential bias [37].

It is interesting to note that some associations between CRC and other health outcomes detected by the sensitivity analysis of including CRC cases were driven by the presence of CRC disease. Although these associations were consistent with the results from DTB (i.e. anaemia, type 2 diabetes mellitus, renal failure, bacterial infection, gonarthrosis, and secondary malignant of lymph node, lung, peritoneum and liver), none was detected by the main analysis when excluding CRC cases. Based on our power estimation, all these phenotypes should have enough power to detect an effect estimate (OR) of less than 1.20. The associations detected after including CRC cases may indicate a shared biological pathway, pre-cancer phenotypes, CRC symptom induced diseases, or post-treatment effects. To further test our findings, we divided the dataset into ten groups based on PRS deciles and found that the case distribution of those phenotypes in the datasets with and without CRC cases was similar. Second, we found that the difference in case proportions in the highest and lowest deciles between the two datasets was similar for those diseases. Therefore, we conclude that these associations should be driven by CRC.

There is evidence for the association between anaemia or markers of anaemia, type 2 diabetes mellitus and specific bacterial species and CRC from observational studies [2, 7, 39–41], but these associations may be consequences of CRC or its treatment. Existing evidence showed that about 50% of CRC patients have anaemia (defined as haemoglobin <12 g/dl in females and <13 g/dl in males) [42]. The chronic blood loss and iron homeostasis defect caused by CRC as well as the subsequent iron deficiency anaemia could explain the decrease of red blood cells among CRC patients [43]. Evidence from observational studies showed that the association between type 2 diabetes mellitus and CRC may be due to shared risk factors, such as insulin resistance, inflammation, hyperglycemia, obesity, physical activity and microbiota [2]. The association with insulin resistance can be driven by obesity [44] and the association with hyperglycemia may be related to diabetic renal complications [41], but we did not identify any associations with obesity or diabetic renal complications in this study. Associations with renal failure have been detected by our sensitivity analysis, which may be due to treatment-related effects including surgical trauma, acute kidney injury and chemotherapy. Nevertheless, we replicated several associations between CRC PRS and cancers in common CRC metastatic sites including secondary cancer in lymph nodes, lung, peritoneum and liver [45].

### Strengths and limitations

In this study, we used the CRC genetic predisposition as exposure and then searched systematically for associations with a wide range of phenotypes defined by ICD codes or a combination of ICD codes (PheCODEs). Being an instrumental variable approach the described PheWAS framework and the use of CRC PRS minimised the influence of reverse causality and confounding effects that are common in observational studies such as the DTB. A PheCODEs based PheWAS conducted as part of the Michigan Genomics Initiative explored associations between several PRSs of CRC and other phenotypes and reported results consistent with our PheWAS analyses [46]. A recent study used the PRS of CRC to scan its association with 15 other cancers and did not find significant associations [47]. In this study, we used recent GWAS findings to construct a genetic measure of CRC and adopted a phenome-wide association framework using the UKBB, a repository with a big enough sample size and high quality, curated, disease record-linkage to national cancer registry, death registry and hospital inpatient systems. The phenome-wide association framework included two methods, which accounted for the differences in scope and structure of phenome scanning and used different methods of ICD hierarchy. Furthermore, to test the robustness of our results, we performed a series of sensitivity analyses.

This study has several limitations. First, although we have performed several sensitivity analyses, we cannot rule out all pleiotropic effects caused by multiple genetic instruments unless all the biological effects of each SNP have been detected. Second, since most of the cases were collected from inpatients, phenotypes that do not usually require hospitalisation could be missed. Third, even though self-reporting health outcomes could have captured milder manifestations of a specific disease or diseases, we did not include self-reported data to minimise any misclassification bias. Reasons such as poor patient-clinician communication, self-diagnosis of patients based on their symptoms, and insufficient medical knowledge among patients may contribute to misclassification bias for self-reported health outcomes [48]. Fourth, considering the potential limitation caused by low power, we restricted our study to phenotypes with more than 20 cases, but limited power still cannot be eliminated for phenotypes with more cases. Finally, our work is limited to studies on white individuals and therefore the generalisability of the conclusions to other populations is uncertain.

## Conclusion

In summary, by taking into account all the findings from PheWAS, TreeWAS, DTB and sensitivity analyses, we found surprisingly few associations linked to CRC genetic predisposition. The only convincing associations were observed between CRC genetic predisposition and benign or pre-malignant colorectal neoplasms, neoplasms of unspecified sites, which are well-established pre-malignant lesions with shared biological pathways. The association with diverticular disease may be due to shared aetiology or biased ascertainment through investigation in those with higher environmental risk factors linked to both conditions.

## Supplementary information

SUPPLEMENTAL MATERIAL

## Data Availability

Not applicable.
