# Supplementary material for: Phenome-wide association study (PheWAS) of colorectal cancer risk SNP effects on health outcomes in UK Biobank
Source: Br J Cancer. 2021 Dec 15;126(5):822–30. doi: 10.1038/s41416-021-01655-9 (PMC8888597; doi:10.1038/s41416-021-01655-9)
Supplement: Supplementary file 1 — SUPPLEMENTAL MATERIAL [file 41416_2021_1655_MOESM1_ESM.docx]

**Phenome-wide association study (PheWAS) of colorectal cancer risk SNP effects on health outcomes in UK Biobank.**

**Running title: PheWAS and TreeWAS of colorectal cancer risk on multiple health outcomes.**

Authors: Xiaomeng Zhang^1^, Xue Li^1,2^, Yazhou He^3,4^, Phillip Law^5^, Susan M Farrington^3^, Harry Campbell^1^, Ian PM Tomlinson^6^, Richard S Houlston^5^, Malcolm G Dunlop^3^, Maria Timofeeva^3,7#^, Evropi Theodoratou^1,6#^

1 Centre for Global Health, Usher Institute, University of Edinburgh, UK

2 School of Public Health and the Second Affiliated Hospital, Zhejiang University, Hangzhou, China

3 Colon Cancer Genetics Group, Cancer Research UK Edinburgh Centre and Medical Research Council Human Genetics Unit, Institute of Genetics and Cancer, University of Edinburgh, Edinburgh, UK

4 Department of Oncology, West China School of Public Health and West China Fourth Hospital, Sichuan University, Chengdu, China

5 Division of Genetics and Epidemiology, The Institute of Cancer Research, London, UK

6 Cancer Research UK Edinburgh Centre, Institute of Genetics and Cancer, University of Edinburgh, Edinburgh, UK

7 Danish Institute for Advanced Study (DIAS), Department of Public Health, University of Southern Denmark, Odense, Denmark

#Correspondence to

Evropi Theodoratou ([e.theodoratou@ed.ac.uk](mailto:e.theodoratou@ed.ac.uk)), Centre for Global Health, Usher Institute, University of Edinburgh, Teviot Place, Edinburgh, United Kingdom

ORCID: 0000-0001-5887-9132

Maria Timofeeva (mtimofeeva@health.sdu.dk), Danish Institute for Advanced Study (D-IAS), Department of Public Health, University of Southern Denmark, J.B. Winsløws Vej 9, DK-5000 Odense C, Denmark

ORCID: 0000-0002-2503-4253

Contents

[Supplementary Methods 3](#_Toc82592947)

[UK Biobank data 3](#_Toc82592948)

[Genotype data 3](#_Toc82592949)

[Phenotype data 3](#_Toc82592950)

[Quality control of participants 3](#_Toc82592951)

[Estimation of variance explained by genetic instrument of CRC risk 3](#_Toc82592952)

[Danish Disease Trajectory Browser 3](#_Toc82592953)

[Genetic correlation and bi-directional Mendelian randomisation (MR) analyses to investigate the association between diverticular disease and colorectal cancer risk 4](#_Toc82592954)

[Supplementary Tables 5](#_Toc82592955)

[Table S1 Colorectal cancer variants used for the generation of polygenic risk score 5](#_Toc82592956)

[Table S2 Characteristics of the datasets for the main analysis and the sensitivity analysis 9](#_Toc82592957)

[Table S3 Relation between generated polygenic risk sores of colorectal cancer and colorectal cancer status in the UK Biobank 9](#_Toc82592958)

[Table S4 The number of PheCODEs or nodes related to the polygenic risk score of colorectal cancer in each disease group 10](#_Toc82592959)

[Table S5 PheWAS results by using colorectal cancer specific polygenic risk score (CRC PRS_53_) in the UK Biobank 11](#_Toc82592960)

[Table S6 TreeWAS results by using colorectal cancer specific polygenic risk score (CRC PRS_53_) in the UK Biobank 11](#_Toc82592961)

[Table S7 PheWAS results by using polygenic risk score of colorectal cancer (CRC PRS_127_) in the UK Biobank with colorectal cancer cases included 12](#_Toc82592962)

[Table S8 TreeWAS results by using polygenic risk score of colorectal cancer (CRC PRS_127_) in the UK Biobank with colorectal cancer cases included 13](#_Toc82592963)

[Table S9 Colorectal cancer associated phenotypes identified by Danish Disease Trajectory Browser 16](#_Toc82592964)

[Table S10 Genetic variants for diverticular disease and colorectal cancer risk 18](#_Toc82592965)

[Table S11 Results of genetic correlation and bi-directional Mendelian randomisation analyses for the association between CRC and diverticular disease. 24](#_Toc82592966)

[Supplementary Figures 25](#_Toc82592967)

[Figure S1 The process of colorectal cancer risk SNPs selection for generating polygenic risk score 25](#_Toc82592968)

[Figure S2 Sample quality control in the UK Biobank 25](#_Toc82592969)

[Figure S3 Proportion of cases in 10 equal deciles of colorectal cancer polygenic risk score (CRC PRS_127_) in UK Biobank with or without colorectal cancer cases 26](#_Toc82592970)

[Reference 31](#_Toc82592971)

# Supplementary Methods

## UK Biobank data

### Genotypic data

Genotyping, quality control and genotype imputation were conducted by the UK Biobank team before the data release and the procedure is described by Bycroft et al^1^. The initial 50,000 participants were genotyped by the Affymetrix UK BiLEVE Axiom array and the remaining 450,000 participants were genotyped by the Affymetrix UK Biobank Axiom array. Genotype imputation was performed using a merged reference panel of the Haplotype Reference Consortium (HRC)^2^ and the UK10K haplotype resources^3^, and the classical allelic variations at the MHC region were further imputed by using an additional multi-population reference panel^4^. For study specific quality control, a list of field variables was made available by the UK Biobank to indicate the genotype quality, population structure, and genetic relatedness.

### Phenotypic data

UK Biobank linked its initial survey with a variety of national health systems and sources to achieve the clinical follow up of the enrolled participants. Currently, three digital health records (i.e., hospital inpatient episodes, cancer registry data and death registry data) have been incorporated. The International Classification of Diseases (ICD) version 9 and 10 (ICD-10 or ICD-9) coding systems from the World Health Organization were applied for the coding of these databases according to the date of the record. Primary and/or secondary ICD codes are available in the hospital inpatient data and/or death registry data to classify the main causes and contributory causes of the event of hospitalization and/or death respectively. The phenotype records of each participant were extracted from any of the three datasets and duplicates were removed. In the phenotype table, each participant had one binary record for each health outcome, which was recorded as *‘TRUE’* (when the participant had the health condition) or *‘FALSE’* (when the participant did not have the health condition). When participants were hospitalised multiple times due to the same reason, they were also recorded as *‘TRUE’* for this phenotype. To avoid the influence of colorectal cancer (CRC) comorbidities, CRC cases were removed from the main analysis. Based on ICD 10, CRC includes malignant neoplasm of caecum (C18.0), ascending colon (C18.2), hepatic flexure (C18.3), transverse colon (C18.4), splenic flexure (C18.5), descending colon (C18.6), sigmoid colon (C18.7), overlapping lesion of colon (C18.8), and colon unspecified (C18.9), malignant neoplasm of rectosigmoid junction (C19), and malignant neoplasm of rectum (C20).

### Quality control of participants

For 488,366 participants in the UK Biobank with genotypic data, 80,177 samples were excluded because of consent withdrawal, non-white British ethnical background, sex mismatch, sex aneuploidy, and high missing rate/outlier. To keep the representative of allele frequencies in the whole population, we retained one participant within each family (among participants with identity by descent [IBD]>0.1875).^5^ Therefore, 68,933 samples were excluded due to kinship inference. The detailed information on genotyping, quality control and genetic imputation were described in a previously published paper.^6^ The process of the sample section is presented in Figure S1.

### Estimation of variance explained by genetic instrument of CRC risk

We estimated the variance of CRC risk explained by the genetic instruments as 30.6% through the following formula ($2\times EAF\times\left( 1-EAF \right)\times\beta^{2}$).^7, 8^ EAF is the effect allele frequency, beta is the effect size of the association between each genetic variant and CRC.

### Danish Disease Trajectory Browser

The Danish Disease Trajectory Browser (<http://dtb.cpr.ku.dk>) is a tool that allows users to explore disease trajectories by ICD-10 codes.^9^ The details of this browser have been described previously.^9^ Briefly, this browser was created based on the Danish National Patient Register (NPR) dataset,^10^ which covered 7.2 million patients during the period between 1994 and 2018. The cause of death within 5 years of a diagnosis was derived from The Danish Register of Causes of Death,^11^ which is an external dataset linked with NPR. To create disease trajectories, the following steps were performed: 1) A total of 10,000 randomly selected individuals were matched for each of the diseases of a diagnosis pair after controlling for age, sex, discharge type and discharge week, in order to identify statistically significant diagnosis pairs within a 5-year time window. The risk ratio was then calculated, and the P values were estimated using a binomial distribution with a Bonferroni corrected P value threshold of 1.21×10^-8^. 2) For the statistically significant diagnosis pairs, the directionality that one disease occurred prior to another disease compared to the opposite was estimated using a binomial distribution with a P value threshold of 0.05. 3) Linear trajectories were built for the significant directional diagnosis pairs. For example, if disease 1 to disease 2 and disease 2 to disease 3 were significant directional diagnosis pairs, the linear trajectories can be built as disease 1 to disease 2 to disease 3. A minimum of 20 patients for each disease was required to build disease trajectories.

### Genetic correlation and bi-directional Mendelian randomisation (MR) analyses to investigate the association between diverticular disease and colorectal cancer risk

To confirm the direction of association between diverticular disease and CRC, we performed a genetic correlation analysis and a bi-directional MR analysis. We extracted 51 and 42 leading variants for diverticular disease from the two most up-to-date genome-wide association studies (GWASs) at P<5×10^-8^ respectively.^12, 13^ A total of 57 variants were retained for analysis after removing 20 duplicates, four missing variants, and 12 variants in linkage disequilibrium (LD) at R^2^>0.2 based on the 1000 Genomes European reference panel. Effect estimates of these variants on CRC were extracted from Law et al after removing UK Biobank (Table S10).^14^ We took the 127 genetic variants for CRC as the instrument of CRC and their effects on the diverticular disease were extracted from Schafmayer et al.^12^ One variant was removed due to missing in Schafmayer et al. A total of 126 genetic variants of CRC were applied to explore the potential causal association between CRC and diverticular disease (Table S10). The correlation analysis was performed based on the 183 genetic variants of diverticular disease and colorectal cancer risk by using the ‘cor.test’ function of R. The causal effects and the corresponding standard errors of exposures on outcomes were calculated by using the random effects inverse variance-weighted method.^15^ MR-Egger was applied to explore any potential bias introduced by pleiotropy. In particular, when the intercept of MR-Egger differs from zero (at p<0.05), then either directional pleiotropy is indicated, or the InSIDE assumption is violated.^16^ All analyses were performed in R (version 3.6.1).

# Supplementary Tables

## Table S1 Colorectal cancer variants used for the generation of polygenic risk score

| Variants | Chr | Position | Effect allele/ other allele | EAF | Effect size extracted from the reference GWASs^#^ | | | Effect size without UK Biobank data | | | Original  publication | Reference | PRS |
| --- | --- | --- | --- | --- | --- | --- | --- | --- | --- | --- | --- | --- | --- |
|  |  |  |  |  | Beta | SE | P | Beta* | SE* | P* |  |  |  |
| rs10049390 | 3 | 133701119 | A/G | 0.71 | 0.058 | 0.010 | 3.79E-09 | 0.058 | 0.014 | 4.90E-05 | New variants | Huyghe et al. 2019 | PRS_127,_ PRS_53_ |
| rs1078643 | 17 | 10707241 | A/G | 0.75 | 0.077 | 0.011 | 6.63E-12 | 0.068 | 0.015 | 9.20E-06 | New variants | Huyghe et al. 2019 | PRS_127,_ PRS_53_ |
| rs10821907 | 10 | 52648454 | C/T | 0.83 | 0.077 | 0.012 | 5.00E-10 | 0.077 | 0.016 | 1.10E-06 | Schmit et al. 2018 | Huyghe et al. 2019 | PRS_127_ |
| rs10980628 | 9 | 113671403 | C/T | 0.21 | 0.068 | 0.011 | 2.77E-09 | 0.049 | 0.013 | 3.07E-04 | New variants | Huyghe et al. 2019 | PRS_127_ |
| rs11087784 | 20 | 7740976 | G/A | 0.15 | 0.086 | 0.011 | 2.70E-13 | 0.086 | 0.016 | 2.50E-07 | Jia et al. 2013 | Huyghe et al. 2019 | PRS_127,_ PRS_53_ |
| rs11196170 | 10 | 114722621 | A/G | 0.22 | 0.058 | 0.011 | 3.30E-07 | 0.058 | 0.014 | 5.70E-05 | Zhang et al. 2014 | Huyghe et al. 2019 | PRS_127_ |
| rs11610543 | 12 | 43134191 | G/A | 0.52 | 0.049 | 0.008 | 1.30E-09 | 0.049 | 0.011 | 1.14E-05 | New variants | Huyghe et al. 2019 | PRS_127,_ PRS_53_ |
| rs11874392 | 18 | 46453156 | A/T | 0.55 | 0.157 | 0.008 | 3.80E-74 | 0.148 | 0.011 | 5.90E-35 | Broderick et al. 2007 | Huyghe et al. 2019 | PRS_127_ |
| rs11884596 | 2 | 199612407 | C/T | 0.39 | 0.058 | 0.010 | 3.62E-09 | 0.049 | 0.014 | 4.63E-04 | New variants | Huyghe et al. 2019 | PRS_127_ |
| rs12144319 | 1 | 55246035 | C/T | 0.29 | 0.068 | 0.010 | 3.27E-11 | 0.068 | 0.014 | 1.35E-06 | New variants | Huyghe et al. 2019 | PRS_127,_ PRS_53_ |
| rs12149163 | 16 | 86339315 | T/C | 0.50 | 0.049 | 0.008 | 5.40E-09 | 0.058 | 0.012 | 1.90E-06 | Schmit et al. 2018 | Huyghe et al. 2019 | PRS_127,_ PRS_53_ |
| rs12246635 | 10 | 114288619 | C/T | 0.10 | 0.095 | 0.013 | 4.90E-12 | 0.077 | 0.020 | 1.30E-04 | Wang et al. 2014 | Huyghe et al. 2019 | PRS_127,_ PRS_53_ |
| rs12372718 | 12 | 51171090 | G/A | 0.39 | 0.086 | 0.008 | 1.90E-23 | 0.086 | 0.012 | 9.60E-13 | Houlston et al. 2010 | Huyghe et al. 2019 | PRS_127_ |
| rs12514517 | 5 | 40280076 | A/G | 0.29 | 0.095 | 0.010 | 3.70E-21 | 0.086 | 0.013 | 3.40E-10 | Schmit et al. 2018 | Huyghe et al. 2019 | PRS_127_ |
| rs12672022 | 7 | 45136423 | T/C | 0.84 | 0.068 | 0.012 | 2.78E-08 | 0.068 | 0.015 | 1.56E-05 | New variants | Huyghe et al. 2019 | PRS_127_ |
| rs13149359 | 4 | 94938618 | A/C | 0.37 | 0.049 | 0.008 | 1.20E-08 | 0.058 | 0.013 | 1.20E-05 | Schmit et al. 2018 | Huyghe et al. 2019 | PRS_127,_ PRS_53_ |
| rs1391441 | 4 | 106128760 | A/G | 0.65 | 0.049 | 0.008 | 1.58E-08 | 0.049 | 0.013 | 1.55E-04 | New variants | Huyghe et al. 2019 | PRS_127_ |
| rs143635270 | 16 | 9242047 | G/A | 0.99 | 0.068 | 0.044 | 1.20E-01 | 0.077 | 0.061 | 2.10E-01 | Al-Tassan et al. 2015 | Huyghe et al. 2019 | PRS_127,_ PRS_53_ |
| rs17011141 | 1 | 222112634 | G/A | 0.21 | 0.086 | 0.010 | 6.10E-16 | 0.086 | 0.014 | 3.60E-09 | Houlston et al. 2010 | Huyghe et al. 2019 | PRS_127_ |
| rs17094983 | 14 | 59189361 | G/A | 0.88 | 0.086 | 0.013 | 4.61E-11 | 0.095 | 0.017 | 8.45E-08 | New variants | Huyghe et al. 2019 | PRS_127,_ PRS_53_ |
| rs174533 | 11 | 61549025 | G/A | 0.67 | 0.068 | 0.010 | 1.20E-11 | 0.077 | 0.012 | 3.50E-10 | Zhang et al. 2014 | Huyghe et al. 2019 | PRS_127_ |
| rs189583 | 20 | 6376457 | G/C | 0.33 | 0.095 | 0.009 | 1.20E-22 | 0.095 | 0.013 | 6.10E-13 | Houlston et al. 2008 | Huyghe et al. 2019 | PRS_127_ |
| rs2186607 | 11 | 101656397 | T/A | 0.50 | 0.049 | 0.008 | 1.47E-09 | 0.049 | 0.011 | 1.11E-05 | New variants | Huyghe et al. 2019 | PRS_127_ |
| rs2250430 | 12 | 6421174 | T/A | 0.71 | 0.068 | 0.011 | 3.30E-10 | 0.068 | 0.014 | 8.80E-07 | Zhang et al. 2014 | Huyghe et al. 2019 | PRS_127,_ PRS_53_ |
| rs2516420 | 6 | 31449620 | C/T | 0.93 | 0.113 | 0.017 | 2.01E-10 | 0.058 | 0.023 | 1.28E-02 | New variants | Huyghe et al. 2019 | PRS_127_ |
| rs2735940 | 5 | 1296486 | G/A | 0.50 | 0.086 | 0.008 | 5.10E-25 | 0.095 | 0.012 | 1.80E-15 | Schmit et al. 2018 | Huyghe et al. 2019 | PRS_127_ |
| rs28488 | 20 | 6762221 | T/C | 0.67 | 0.068 | 0.009 | 2.60E-14 | 0.077 | 0.013 | 2.34E-09 | New variants | Huyghe et al. 2019 | PRS_127_ |
| rs28840750 | 19 | 33519927 | T/G | 0.95 | 0.191 | 0.018 | 3.70E-23 | 0.199 | 0.025 | 2.30E-14 | Houlston et al. 2008 | Huyghe et al. 2019 | PRS_127_ |
| rs3125049 | 6 | 160754712 | G/A | 0.85 | 0.020 | 0.010 | 5.10E-02 | 0.020 | 0.022 | 3.70E-01 | Cui et al. 2010 | Huyghe et al. 2019 | PRS_127_ |
| rs3133285 | 8 | 117629411 | G/C | 0.82 | 0.068 | 0.011 | 6.00E-10 | 0.068 | 0.015 | 4.30E-06 | Zeng et al. 2016 | Huyghe et al. 2019 | PRS_127,_ PRS_53_ |
| rs3217810 | 12 | 4388271 | T/C | 0.13 | 0.122 | 0.013 | 3.60E-19 | 0.148 | 0.020 | 7.10E-13 | Whiffin et al. 2014 | Huyghe et al. 2019 | PRS_127,_ PRS_53_ |
| rs3217874 | 12 | 4400808 | T/C | 0.43 | 0.058 | 0.010 | 2.38E-09 | 0.049 | 0.013 | 2.07E-04 | New variants | Huyghe et al. 2019 | PRS_127_ |
| rs34405347 | 9 | 101679752 | T/G | 0.90 | 0.086 | 0.015 | 3.06E-08 | 0.077 | 0.019 | 5.54E-05 | New variants | Huyghe et al. 2019 | PRS_127,_ PRS_53_ |
| rs34797592 | 19 | 16417198 | T/C | 0.11 | 0.086 | 0.013 | 4.15E-10 | 0.086 | 0.019 | 8.24E-06 | New variants | Huyghe et al. 2019 | PRS_127_ |
| rs35107139 | 14 | 54419106 | C/A | 0.42 | 0.086 | 0.008 | 1.80E-22 | 0.086 | 0.012 | 2.80E-12 | Houlston et al. 2008 | Huyghe et al. 2019 | PRS_127_ |
| rs35470271 | 3 | 40915239 | G/A | 0.15 | 0.095 | 0.011 | 1.20E-16 | 0.122 | 0.015 | 1.60E-14 | Schumacher et al. 2015 | Huyghe et al. 2019 | PRS_127,_ PRS_53_ |
| rs35808169 | 12 | 4368607 | C/T | 0.17 | 0.077 | 0.011 | 1.50E-11 | 0.095 | 0.015 | 6.90E-10 | Jia et al. 2013 | Huyghe et al. 2019 | PRS_127_ |
| rs4313119 | 8 | 128571855 | G/T | 0.75 | 0.058 | 0.010 | 2.13E-09 | 0.077 | 0.014 | 3.25E-08 | New variants | Huyghe et al. 2019 | PRS_127,_ PRS_53_ |
| rs448513 | 2 | 159964552 | C/T | 0.35 | 0.049 | 0.009 | 4.44E-08 | 0.058 | 0.013 | 1.93E-05 | New variants | Huyghe et al. 2019 | PRS_127_ |
| rs4759277 | 12 | 57533690 | A/C | 0.33 | 0.049 | 0.008 | 9.38E-09 | 0.068 | 0.014 | 8.37E-07 | New variants | Huyghe et al. 2019 | PRS_127_ |
| rs4917993 | 10 | 104791595 | C/G | 0.87 | 0.049 | 0.013 | 1.70E-04 | 0.068 | 0.018 | 1.90E-04 | Zeng et al. 2016 | Huyghe et al. 2019 | PRS_127_ |
| rs4968127 | 17 | 809643 | G/A | 0.37 | 0.068 | 0.009 | 1.30E-12 | 0.058 | 0.012 | 6.90E-07 | Zhang et al. 2014 | Huyghe et al. 2019 | PRS_127,_ PRS_53_ |
| rs4976270 | 5 | 134467220 | C/T | 0.55 | 0.068 | 0.008 | 4.80E-15 | 0.068 | 0.011 | 3.70E-09 | Jia et al. 2013 | Huyghe et al. 2019 | PRS_127_ |
| rs55990915 | 12 | 117763309 | A/C | 0.13 | 0.068 | 0.014 | 1.80E-06 | 0.095 | 0.019 | 9.50E-07 | Schumacher et al. 2015 | Huyghe et al. 2019 | PRS_127,_ PRS_53_ |
| rs56324967 | 15 | 67402824 | C/T | 0.65 | 0.068 | 0.009 | 1.14E-13 | 0.068 | 0.013 | 2.24E-07 | New variants | Huyghe et al. 2019 | PRS_127,_ PRS_53_ |
| rs58658771 | 15 | 33001734 | A/T | 0.19 | 0.140 | 0.010 | 6.00E-37 | 0.122 | 0.015 | 3.80E-15 | Tomlinson et al. 2011 | Huyghe et al. 2019 | PRS_127_ |
| rs597808 | 12 | 111973358 | G/A | 0.52 | 0.077 | 0.009 | 2.60E-16 | 0.086 | 0.013 | 5.10E-11 | Schumacher et al. 2015 | Huyghe et al. 2019 | PRS_127_ |
| rs6063514 | 20 | 49055318 | C/T | 0.61 | 0.068 | 0.009 | 7.60E-13 | 0.068 | 0.013 | 2.20E-07 | Schmit et al. 2018 | Huyghe et al. 2019 | PRS_127_ |
| rs62396735 | 6 | 41702582 | C/T | 0.29 | 0.030 | 0.009 | 6.30E-04 | 0.030 | 0.014 | 3.60E-02 | Zeng et al. 2016 | Huyghe et al. 2019 | PRS_127_ |
| rs62404966 | 6 | 55712124 | C/T | 0.76 | 0.058 | 0.010 | 2.60E-09 | 0.058 | 0.013 | 1.70E-05 | Schmit et al. 2018 | Huyghe et al. 2019 | PRS_127,_ PRS_53_ |
| rs6678517 | 1 | 183002639 | A/G | 0.59 | 0.077 | 0.009 | 2.40E-16 | 0.049 | 0.012 | 2.90E-05 | Whiffin et al. 2014 | Huyghe et al. 2019 | PRS_127_ |
| rs6781752 | 3 | 66365163 | A/G | 0.21 | 0.058 | 0.011 | 7.10E-08 | 0.068 | 0.015 | 6.90E-06 | Schumacher et al. 2015 | Huyghe et al. 2019 | PRS_127,_ PRS_53_ |
| rs6983267 | 8 | 128413305 | G/T | 0.52 | 0.148 | 0.008 | 3.40E-64 | 0.140 | 0.012 | 1.50E-30 | Tomlinson et al. 2007 | Huyghe et al. 2019 | PRS_127_ |
| rs704017 | 10 | 80819132 | G/A | 0.58 | 0.077 | 0.009 | 5.20E-18 | 0.077 | 0.012 | 6.20E-10 | Zhang et al. 2014 | Huyghe et al. 2019 | PRS_127_ |
| rs7121958 | 11 | 74280012 | G/T | 0.51 | 0.077 | 0.008 | 1.40E-20 | 0.086 | 0.012 | 2.60E-12 | Dunlop et al. 2012 | Huyghe et al. 2019 | PRS_127_ |
| rs7160450 | 14 | 54457231 | T/C | 0.38 | 0.058 | 0.009 | 4.00E-10 | 0.049 | 0.013 | 1.20E-04 | Tomlinson et al. 2011 | Huyghe et al. 2019 | PRS_127,_ PRS_53_ |
| rs72942485 | 3 | 112999560 | G/A | 0.98 | 0.174 | 0.030 | 2.08E-08 | 0.148 | 0.040 | 2.49E-04 | New variants | Huyghe et al. 2019 | PRS_127,_ PRS_53_ |
| rs7300312 | 12 | 115890922 | C/T | 0.57 | 0.068 | 0.009 | 7.50E-14 | 0.068 | 0.012 | 1.60E-08 | Schmit et al. 2018 | Huyghe et al. 2019 | PRS_127_ |
| rs73068325 | 19 | 59079096 | T/C | 0.18 | 0.068 | 0.012 | 4.25E-08 | 0.058 | 0.016 | 2.11E-04 | New variants | Huyghe et al. 2019 | PRS_127_ |
| rs7333607 | 13 | 37462010 | G/A | 0.22 | 0.077 | 0.010 | 6.32E-13 | 0.086 | 0.015 | 2.46E-08 | New variants | Huyghe et al. 2019 | PRS_127_ |
| rs75610640 | 2 | 192562764 | C/T | 0.16 | 0.039 | 0.012 | 1.00E-03 | 0.049 | 0.017 | 4.30E-03 | Peters et al. 2013 | Huyghe et al. 2019 | PRS_127,_ PRS_53_ |
| rs75954926 | 17 | 81061048 | G/A | 0.64 | 0.086 | 0.010 | 2.96E-18 | 0.095 | 0.014 | 9.36E-11 | New variants | Huyghe et al. 2019 | PRS_127_ |
| rs7708610 | 5 | 40102443 | A/G | 0.35 | 0.058 | 0.010 | 3.78E-09 | 0.049 | 0.013 | 1.32E-04 | New variants | Huyghe et al. 2019 | PRS_127,_ PRS_53_ |
| rs78341008 | 13 | 73791554 | C/T | 0.07 | 0.113 | 0.018 | 3.21E-10 | 0.122 | 0.025 | 1.41E-06 | New variants | Huyghe et al. 2019 | PRS_127,_ PRS_53_ |
| rs9271695 | 6 | 32593080 | G/A | 0.81 | 0.086 | 0.011 | 1.12E-13 | 0.086 | 0.016 | 1.32E-07 | New variants | Huyghe et al. 2019 | PRS_127_ |
| rs983318 | 17 | 70413253 | A/G | 0.24 | 0.058 | 0.010 | 5.57E-09 | 0.068 | 0.014 | 1.15E-06 | New variants | Huyghe et al. 2019 | PRS_127,_ PRS_53_ |
| rs983402 | 2 | 199781586 | T/C | 0.30 | 0.068 | 0.010 | 7.71E-12 | 0.049 | 0.012 | 7.17E-05 | New variants | Huyghe et al. 2019 | PRS_127_ |
| rs9924886 | 16 | 68743939 | A/C | 0.73 | 0.058 | 0.010 | 3.10E-08 | 0.058 | 0.014 | 3.40E-05 | Houlston et al. 2008 | Huyghe et al. 2019 | PRS_127_ |
| rs994308 | 20 | 6603622 | C/T | 0.58 | 0.058 | 0.008 | 8.57E-12 | 0.049 | 0.012 | 8.19E-05 | New variants | Huyghe et al. 2019 | PRS_127_ |
| rs10152518 | 15 | 68177162 | G/A | 0.19 | 0.077 | 0.014 | 3.24E-08 | 0.074 | 0.016 | 2.20E-06 | New variants | Law et al. 2019 | PRS_127_ |
| rs10161980 | 13 | 34093518 | C/G | 0.62 | 0.058 | 0.010 | 1.96E-08 | 0.071 | 0.013 | 6.44E-08 | Schmit SL, et al, 2018 | Law et al. 2019 | PRS_127,_ PRS_53_ |
| rs10849432 | 12 | 6385727 | T/C | 0.90 | 0.058 | 0.017 | 6.89E-04 | 0.065 | 0.021 | 2.10E-03 | Zhang B, et al, 2014 | Law et al. 2019 | PRS_127,_ PRS_53_ |
| rs10849438 | 12 | 6412036 | G/T | 0.12 | 0.113 | 0.017 | 1.04E-10 | 0.107 | 0.026 | 3.98E-05 | Zhang B, et al, 2014 | Law et al. 2019 | PRS_127,_ PRS_53_ |
| rs10951878 | 7 | 46926695 | C/T | 0.49 | 0.058 | 0.010 | 1.10E-08 | 0.051 | 0.012 | 2.57E-05 | New variants | Law et al. 2019 | PRS_127_ |
| rs11692435 | 2 | 98275354 | G/A | 0.90 | 0.113 | 0.019 | 1.22E-08 | 0.105 | 0.028 | 2.20E-04 | New variants | Law et al. 2019 | PRS_127_ |
| rs12522693 | 5 | 130195731 | G/A | 0.85 | 0.010 | 0.011 | 3.51E-01 | -0.014 | 0.019 | 4.70E-01 | Jiang K, et al, 2015 | Law et al. 2019 | PRS_127_ |
| rs12603526 | 17 | 800593 | C/T | 0.02 | 0.049 | 0.034 | 1.54E-01 | 0.004 | 0.041 | 9.16E-01 | Zhang B, et al, 2014 | Law et al. 2019 | PRS_127,_ PRS_53_ |
| rs12635946 | 3 | 112916918 | C/T | 0.62 | 0.077 | 0.011 | 1.02E-11 | 0.089 | 0.013 | 4.16E-12 | New variants | Law et al. 2019 | PRS_127,_ PRS_53_ |
| rs12979278 | 19 | 49218602 | T/C | 0.53 | 0.068 | 0.011 | 6.11E-10 | 0.075 | 0.015 | 4.50E-07 | New variants | Law et al. 2019 | PRS_127_ |
| rs13020391 | 2 | 219184436 | C/T | 0.63 | 0.086 | 0.011 | 2.47E-13 | 0.082 | 0.013 | 2.78E-10 | Orlando O, et al, 2016 | Law et al. 2019 | PRS_127_ |
| rs1321310 | 6 | 36623124 | C/T | 0.24 | 0.086 | 0.013 | 5.80E-11 | 0.088 | 0.014 | 7.65E-10 | Dunlop MG, et al, 2012 | Law et al. 2019 | PRS_127,_ PRS_53_ |
| rs1330889 | 13 | 78,609,615 | C/T | 0.87 | 0.104 | 0.017 | 6.50E-10 | 0.101 | 0.016 | 6.50E-10 | New variants | Law et al. 2019 | PRS_127_ |
| rs1412834 | 9 | 22110131 | T/C | 0.50 | 0.077 | 0.010 | 4.13E-14 | 0.096 | 0.012 | 4.56E-15 | New variants | Law et al. 2019 | PRS_127_ |
| rs16878812 | 6 | 35569562 | A/G | 0.89 | 0.104 | 0.017 | 1.67E-09 | 0.105 | 0.020 | 1.32E-07 | Schmit SL,et al, 2018 | Law et al. 2019 | PRS_127_ |
| rs16892766 | 8 | 117630683 | C/A | 0.09 | 0.231 | 0.018 | 1.21E-35 | 0.231 | 0.021 | 7.35E-28 | Tomlinson IP, et al, 2008 | Law et al. 2019 | PRS_127_ |
| rs16969681 | 15 | 32993111 | T/C | 0.09 | 0.095 | 0.009 | 2.85E-24 | 0.192 | 0.031 | 1.01E-09 | New variants | Law et al. 2019 | PRS_127_ |
| rs17035289 | 4 | 106048291 | T/C | 0.83 | 0.095 | 0.015 | 2.73E-10 | 0.101 | 0.016 | 7.03E-10 | New variants | Law et al. 2019 | PRS_127_ |
| rs1741640 | 20 | 60932414 | C/T | 0.77 | 0.148 | 0.013 | 2.09E-28 | 0.165 | 0.016 | 5.38E-25 | Houlston RS, et al, 2010 | Law et al. 2019 | PRS_127_ |
| rs17816465 | 15 | 33156386 | A/G | 0.20 | 0.104 | 0.013 | 8.36E-15 | 0.092 | 0.023 | 6.72E-05 | New variants | Law et al. 2019 | PRS_127,_ PRS_53_ |
| rs17836917 | 17 | 32047282 | G/A | 0.98 | 0.068 | 0.058 | 2.47E-01 | 0.057 | 0.071 | 4.21E-01 | Jiang K, et al, 2015 | Law et al. 2019 | PRS_127,_ PRS_53_ |
| rs186722897 | 4 | 163374639 | T/A | 0.05 | 0.095 | 0.031 | 2.01E-03 | 0.093 | 0.030 | 2.01E-03 | Schmit SL, et al, 2014 | Law et al. 2019 | PRS_127,_ PRS_53_ |
| rs2070699 | 6 | 12292772 | T/G | 0.48 | 0.068 | 0.011 | 3.88E-09 | 0.056 | 0.017 | 1.03E-03 | New variants | Law et al. 2019 | PRS_127_ |
| rs2179593 | 20 | 42660286 | A/C | 0.72 | 0.068 | 0.011 | 4.62E-09 | 0.063 | 0.014 | 4.81E-06 | New variants | Law et al. 2019 | PRS_127_ |
| rs2193352 | 10 | 101346609 | G/A | 0.19 | 0.104 | 0.013 | 1.74E-15 | 0.112 | 0.015 | 2.40E-13 | Whiffin N, et al, 2014 | Law et al. 2019 | PRS_127,_ PRS_53_ |
| rs2295444 | 20 | 33173883 | C/T | 0.50 | 0.020 | 0.009 | 2.57E-02 | 0.020 | 0.012 | 9.65E-02 | Schmit SL, et al, 2018 | Law et al. 2019 | PRS_127_ |
| rs3087967 | 11 | 111156836 | T/C | 0.30 | 0.140 | 0.011 | 6.01E-33 | 0.146 | 0.017 | 3.74E-17 | Tenesa A, et al, 2008 | Law et al. 2019 | PRS_127,_ PRS_53_ |
| rs3131043 | 6 | 30758466 | G/A | 0.43 | 0.068 | 0.012 | 2.67E-08 | 0.050 | 0.028 | 8.07E-02 | New variants | Law et al. 2019 | PRS_127_ |
| rs35446936 | 3 | 169486508 | G/A | 0.70 | 0.068 | 0.012 | 6.12E-08 | 0.073 | 0.019 | 9.33E-05 | Houlston RS, et al, 2010 | Law et al. 2019 | PRS_127_ |
| rs3787089 | 20 | 62316630 | C/T | 0.32 | 0.068 | 0.011 | 5.80E-09 | 0.067 | 0.014 | 1.44E-06 | New variants | Law et al. 2019 | PRS_127_ |
| rs3801081 | 7 | 47511161 | G/A | 0.68 | 0.077 | 0.011 | 2.00E-11 | 0.071 | 0.021 | 8.99E-04 | New variants | Law et al. 2019 | PRS_127,_ PRS_53_ |
| rs3987 | 4 | 118759055 | G/A | 0.38 | 0.020 | 0.012 | 9.74E-02 | 0.012 | 0.013 | 3.56E-01 | Real LM, et al, 2014 | Law et al. 2019 | PRS_127,_ PRS_53_ |
| rs4450168 | 11 | 10286755 | C/A | 0.17 | 0.095 | 0.016 | 1.24E-08 | 0.094 | 0.019 | 7.27E-07 | New variants | Law et al. 2019 | PRS_127_ |
| rs45597035 | 13 | 73649152 | A/G | 0.64 | 0.077 | 0.012 | 2.16E-10 | 0.074 | 0.013 | 1.26E-08 | New variants | Law et al. 2019 | PRS_127_ |
| rs4776316 | 15 | 67007813 | A/G | 0.73 | 0.077 | 0.013 | 1.11E-08 | 0.078 | 0.019 | 3.82E-05 | New variants | Law et al. 2019 | PRS_127_ |
| rs4811050 | 20 | 48980670 | A/G | 0.18 | 0.086 | 0.014 | 4.07E-09 | 0.101 | 0.019 | 1.39E-07 | New variants | Law et al. 2019 | PRS_127_ |
| rs4944940 | 11 | 74415252 | G/A | 0.96 | 0.039 | 0.004 | 3.21E-17 | 0.269 | 0.037 | 2.95E-13 | New variants | Law et al. 2019 | PRS_127_ |
| rs6065668 | 20 | 42532821 | C/T | 0.74 | 0.020 | 0.015 | 1.79E-01 | -0.014 | 0.014 | 3.32E-01 | Tanikawa C, et al, 2018 | Law et al. 2019 | PRS_127,_ PRS_53_ |
| rs6066825 | 20 | 47340117 | A/G | 0.65 | 0.095 | 0.011 | 3.82E-17 | 0.089 | 0.013 | 7.02E-12 | New variants | Law et al. 2019 | PRS_127,_ PRS_53_ |
| rs6085661 | 20 | 6693128 | T/C | 0.39 | 0.086 | 0.011 | 2.95E-15 | 0.088 | 0.012 | 1.45E-12 | New variants | Law et al. 2019 | PRS_127_ |
| rs6091213 | 20 | 49384745 | C/T | 0.26 | 0.077 | 0.012 | 5.68E-10 | 0.077 | 0.016 | 1.12E-06 | New variants | Law et al. 2019 | PRS_127,_ PRS_53_ |
| rs61336918 | 16 | 80007266 | A/T | 0.29 | 0.086 | 0.012 | 2.04E-12 | 0.094 | 0.014 | 1.57E-11 | New variants | Law et al. 2019 | PRS_127,_ PRS_53_ |
| rs61776719 | 1 | 38461319 | C/A | 0.45 | 0.068 | 0.010 | 2.19E-10 | 0.070 | 0.014 | 5.93E-07 | New variants | Law et al. 2019 | PRS_127_ |
| rs6928864 | 6 | 105966894 | C/A | 0.91 | 0.122 | 0.021 | 1.37E-08 | 0.126 | 0.022 | 1.37E-08 | New variants | Law et al. 2019 | PRS_127,_ PRS_53_ |
| rs6933790 | 6 | 41672769 | T/C | 0.83 | 0.095 | 0.015 | 3.65E-10 | 0.085 | 0.021 | 6.77E-05 | Zeng C, et al, 2016 | Law et al. 2019 | PRS_127,_ PRS_53_ |
| rs72647484 | 1 | 22587728 | T/C | 0.91 | 0.122 | 0.019 | 2.20E-10 | 0.122 | 0.030 | 5.20E-05 | Al-Tassan NA, et al, 2015 | Law et al. 2019 | PRS_127,_ PRS_53_ |
| rs73975588 | 17 | 816741 | A/C | 0.87 | 0.095 | 0.016 | 8.71E-09 | 0.103 | 0.026 | 6.00E-05 | Zhang B, et al, 2014 | Law et al. 2019 | PRS_127,_ PRS_53_ |
| rs7398375 | 12 | 57540848 | C/G | 0.72 | 0.086 | 0.013 | 3.91E-10 | 0.089 | 0.017 | 1.96E-07 | New variants | Law et al. 2019 | PRS_127_ |
| rs7495132 | 15 | 91172901 | T/C | 0.12 | 0.104 | 0.017 | 7.92E-10 | 0.112 | 0.025 | 8.69E-06 | New variants | Law et al. 2019 | PRS_127_ |
| rs75686861 | 4 | 145621328 | A/G | 0.10 | 0.113 | 0.018 | 1.76E-09 | 0.118 | 0.021 | 1.89E-08 | New variants | Law et al. 2019 | PRS_127_ |
| rs77776598 | 5 | 1240998 | C/T | 0.06 | 0.049 | 0.008 | 2.84E-10 | 0.144 | 0.027 | 7.25E-08 | New variants | Law et al. 2019 | PRS_127,_ PRS_53_ |
| rs7894531 | 10 | 8734761 | G/A | 0.69 | 0.122 | 0.012 | 4.49E-24 | 0.125 | 0.014 | 2.41E-20 | Tomlinson IP, et al, 2008 | Law et al. 2019 | PRS_127,_ PRS_53_ |
| rs7993934 | 13 | 111074915 | T/C | 0.65 | 0.077 | 0.011 | 3.03E-11 | 0.070 | 0.013 | 5.24E-08 | New variants | Law et al. 2019 | PRS_127,_ PRS_53_ |
| rs812481 | 3 | 66442435 | G/C | 0.52 | 0.010 | 0.013 | 4.38E-01 | 0.006 | 0.012 | 6.01E-01 | Schumacher FR, et al, 2015 | Law et al. 2019 | PRS_127_ |
| rs847208 | 16 | 86254051 | A/C | 0.63 | 0.010 | 0.015 | 5.26E-01 | 0.014 | 0.018 | 4.50E-01 | Tanikawa C, et al, 2018 | Law et al. 2019 | PRS_127,_ PRS_53_ |
| rs899244 | 16 | 86700030 | T/C | 0.21 | 0.086 | 0.013 | 1.13E-10 | 0.089 | 0.018 | 6.12E-07 | New variants | Law et al. 2019 | PRS_127,_ PRS_53_ |
| rs9797885 | 19 | 41873001 | G/A | 0.71 | 0.077 | 0.012 | 2.77E-10 | 0.072 | 0.013 | 9.93E-08 | Zhang B, et al, 2014 | Law et al. 2019 | PRS_127_ |
| rs9831861 | 3 | 53088285 | G/T | 0.59 | 0.068 | 0.011 | 4.17E-10 | 0.061 | 0.012 | 7.39E-07 | New variants | Law et al. 2019 | PRS_127_ |
| EAF: effect allele frequency, SE: standard error, GWAS: genome-wide association study. PRS: polygenic risk score, PRS_127_: PRS generated by 127 genetic variants extracted from two recent GWAS, PRS_53_: PRS generated by excluding 74 genetic variants that were also associated with other phenotypes from searching NHGRI-EBI GWAS Catalog and PhenoScanner.  #: all the effect estimates were extracted from either Huyghe et al. 2019 or Law et al. 2019.  *: estimations after removing UK Biobank data. | | | | | | | | | | | | | |

## Table S2 Characteristics of the datasets for the main analysis and the sensitivity analysis

|  | Full dataset | Excluding CRC cases |
| --- | --- | --- |
| Number of participants | 339,256 | 334,385 |
| Age | 56.9 (SD=8.0) | 56.8 (SD=8.0) |
| Gender | Male 157,135 (46.3%) | Male 154,341 (46.2%) |
|  | Female 182,121 (53.7%) | Female 180,044 (53.8%) |
| BMI (kg/m^2^) | 27.4 (SD=4.8) | 27.4 (SD=4.8) |
| CRC: colorectal cancer, SD: standard deviation, BMI: body mass index. | | |

## Table S3 Association between generated polygenic risk sores of colorectal cancer and colorectal cancer status in the UK Biobank

| Genetic instruments | Total number (number of cases) | Logistic regression | | Q4vsQ1 | |
| --- | --- | --- | --- | --- | --- |
|  |  | OR (95%CI) | P | OR (95%CI) | P |
| PRS_127_ | 339256 (4871) | 1.58 (1.54, 1.63) | 8.32×10^-218^ | 3.07 (2.81,3.36) | 1.40×10^-146^ |
| PRS_53_ |  | 1.29 (1.25, 1.33) | 3.71×10^-70^ | 1.96 (1.80,2.13) | 6.13×10^-57^ |
| CRC: colorectal cancer, PRS: polygenic risk score of CRC, PRS_127_: PRS generated by 127 genetic variants extracted from two recent GWAS, PRS_53_: PRS generated by excluding 74 genetic variants that were also associated with other phenotypes from searching NHGRI-EBI GWAS Catalog and PhenoScanner, logistic regression: the PRS in logistic regression was standardized based on the sample mean and sample deviation; age and sex were adjusted in the logistic regression, Q4vsQ1: odds ratios of the case frequency difference between the top risk quartile to the bottom risk quartile of CRC and chi-square test was performed to calculate the P value. | | | | | |

## Table S4 The number of PheCODEs or nodes related to the polygenic risk score of colorectal cancer in each disease group

| **PheWAS** | | | | |
| --- | --- | --- | --- | --- |
| **Disease groups** | **Number of PheCODEs** | **Number of PheCODEs associated with CRC PRS** | | |
|  |  | **PRS_127_** | **PRS_127_*** | **PRS_53_** |
| Circulatory system | 140 | 0 | 1 | 0 |
| Digestive | 144 | 3 | 10 | 3 |
| Hematopoietic | 41 | 0 | 2 | 0 |
| Musculoskeletal | 111 | 0 | 2 | 0 |
| Neoplasms | 128 | 5 | 15 | 3 |
| Neurological | 71 | 0 | 1 | 0 |
| **TreeWAS** | | | | |
| **Disease groups** | **Number of nodes** | **Number of nodes associated with CRC PRS** | | |
|  |  | **PRS_127_**  **Log_10_(BF_tree_)=669** | **PRS_127_* Log_10_(BF_tree_)=1292** | **PRS_53_**  **Log_10_(BF_tree_)=191** |
| Chapter I Certain infectious and parasitic diseases | 119 | 0 | 5 | 0 |
| Chapter II Neoplasms | 653 | 15 | 46 | 8 |
| Chapter III Diseases of the blood and blood-forming organs and certain disorders involving the immune mechanism | 110 | 0 | 5 | 0 |
| Chapter IV Endocrine nutritional and metabolic diseases | 197 | 0 | 1 | 0 |
| Chapter X Diseases of the respiratory system | 215 | 0 | 2 | 0 |
| Chapter XI Diseases of the digestive system | 418 | 7 | 32 | 12 |
| Chapter XII Diseases of the skin and subcutaneous tissue | 199 | 0 | 2 | 0 |
| Chapter XIII Diseases of the musculoskeletal system and connective tissue | 1245 | 0 | 7 | 0 |
| Chapter XIV Diseases of the genitourinary system | 357 | 0 | 5 | 0 |
| CRC: colorectal cancer, PRS: polygenic risk score of CRC, *: CRC cases were included in the dataset, PRS_127_: PRS generated by 127 genetic variants extracted from two recent GWAS, PRS_53_: PRS generated by 53 genetic variants that were only detected to be associated with CRC. | | | | |

## Table S5 PheWAS results when using the colorectal cancer specific polygenic risk score (CRC PRS_53_) in the UK Biobank

| Description | Group | Number of participants | Number of cases | Beta | SE | FDR q |
| --- | --- | --- | --- | --- | --- | --- |
| Benign neoplasm of colon | neoplasms | 334134 | 18796 | 0.44 | 0.02 | 4.71×10^-79^ |
| Benign neoplasm of unspecified sites | neoplasms | 334385 | 40252 | 0.17 | 0.02 | 1.82×10^-23^ |
| Anal and rectal polyp | digestive | 268006 | 6807 | 0.31 | 0.04 | 4.37×10^-14^ |
| Other disorders of intestine | digestive | 332441 | 71242 | 0.10 | 0.01 | 2.00×10^-12^ |
| Diverticulosis | digestive | 298598 | 27266 | 0.15 | 0.02 | 1.22×10^-11^ |
| Malignant neoplasm, other | neoplasms | 328419 | 91309 | 0.07 | 0.01 | 1.22×10^-7^ |
| SE: standard error, FDR: false discovery rate | | | | | | |

## Table S6 TreeWAS results when using the colorectal cancer specific polygenic risk score (CRC PRS_53_) in the UK Biobank

| Meaning | max_b | b_ci_lhs | b_ci_rhs | POST_ACTIVE |
| --- | --- | --- | --- | --- |
| D12 Benign neoplasm of colon, rectum, anus and anal canal | 0.61 | 0.57 | 0.66 | 1.00 |
| D12.0 Caecum | 0.61 | 0.57 | 0.66 | 1.00 |
| D12.2 Ascending colon | 0.61 | 0.57 | 0.66 | 1.00 |
| D12.3 Transverse colon | 0.61 | 0.57 | 0.66 | 1.00 |
| D12.4 Descending colon | 0.61 | 0.57 | 0.66 | 1.00 |
| D12.5 Sigmoid colon | 0.61 | 0.57 | 0.66 | 1.00 |
| D12.6 Colon, unspecified | 0.61 | 0.57 | 0.66 | 1.00 |
| D12.8 Rectum | 0.61 | 0.57 | 0.66 | 1.00 |
| Chapter XI Diseases of the digestive system | 0.12 | 0.10 | 0.16 | 0.98 |
| K00-K14 Diseases of oral cavity, salivary glands and jaws | 0.12 | 0.10 | 0.16 | 0.98 |
| K08 Other disorders of teeth and supporting structures | 0.12 | 0.10 | 0.16 | 0.96 |
| K50-K52 Noninfective enteritis and colitis | 0.12 | 0.10 | 0.16 | 0.96 |
| K55-K64 Other diseases of intestines | 0.12 | 0.08 | 0.16 | 1.00 |
| K57 Diverticular disease of intestine | 0.12 | 0.10 | 0.16 | 1.00 |
| K57.3 Diverticular disease of large intestine without perforation or abscess | 0.12 | 0.10 | 0.16 | 1.00 |
| K57.9 Diverticular disease of intestine, part unspecified, without perforation or abscess | 0.12 | 0.10 | 0.16 | 1.00 |
| K62.1 Rectal polyp | 0.34 | 0.11 | 0.42 | 1.00 |
| K63 Other diseases of intestine | 0.12 | 0.08 | 0.40 | 0.98 |
| K63.5 Polyp of colon | 0.39 | 0.34 | 0.45 | 1.00 |
| K80-K87 Disorders of gallbladder, biliary tract and pancreas | 0.12 | 0.10 | 0.16 | 0.97 |
| max_b: maximum a posteriori effect estimates (beta) and the 95% credible interval (max_b_lhs, max_b_rhs), POST_ACTIVE: posterior probability for the beta (β) estimate in the tree analysis not being zero. | | | | |

## Table S7 PheWAS results when using the polygenic risk score of colorectal cancer (CRC PRS_127_) in the UK Biobank with colorectal cancer cases included

| Description | Group | Number of participants | Number of cases | Beta | SE | FDR q |
| --- | --- | --- | --- | --- | --- | --- |
| Benign neoplasm of colon | neoplasms | 336189 | 20851 | 0.52 | 0.01 | 0 |
| Colon cancer | neoplasms | 315525 | 3615 | 0.84 | 0.03 | 4.52E-167 |
| Malignant neoplasm of other and ill-defined sites within the digestive organs and peritoneum | neoplasms | 327526 | 5203 | 0.63 | 0.03 | 1.46E-135 |
| Benign neoplasm of unspecified sites | neoplasms | 339256 | 42035 | 0.21 | 0.01 | 1.50E-108 |
| Malignant neoplasm of rectum, rectosigmoid junction, and anus | neoplasms | 314473 | 2563 | 0.80 | 0.04 | 1.10E-107 |
| Anal and rectal polyp | digestive | 270110 | 7479 | 0.41 | 0.02 | 2.91E-81 |
| Malignant neoplasm, other | neoplasms | 333289 | 96125 | 0.13 | 0.01 | 8.38E-71 |
| Other disorders of intestine | digestive | 337174 | 74543 | 0.11 | 0.01 | 4.29E-50 |
| Diverticulosis | digestive | 301755 | 28514 | 0.14 | 0.01 | 3.01E-33 |
| Ileostomy status | digestive | 274839 | 1598 | 0.49 | 0.04 | 4.25E-25 |
| Neoplasm of unspecified nature of digestive system | neoplasms | 323633 | 1310 | 0.53 | 0.05 | 1.12E-24 |
| Secondary malignancy of lymph nodes | neoplasms | 242682 | 5518 | 0.21 | 0.02 | 1.95E-15 |
| Secondary malignant neoplasm of liver | neoplasms | 240115 | 2951 | 0.28 | 0.03 | 6.41E-15 |
| Chemotherapy | neoplasms | 255521 | 18357 | 0.12 | 0.01 | 7.21E-15 |
| Colorectal cancer | neoplasms | 312155 | 245 | 0.89 | 0.11 | 5.08E-13 |
| Secondary malignancy of respiratory organs | neoplasms | 239730 | 2566 | 0.22 | 0.04 | 2.51E-08 |
| Gastrointestinal complications | digestive | 263165 | 534 | 0.45 | 0.08 | 6.64E-07 |
| Other disorders of peritoneum | digestive | 266750 | 4119 | 0.16 | 0.03 | 8.63E-07 |
| Other intestinal obstruction | digestive | 276696 | 3455 | 0.15 | 0.03 | 6.31E-05 |
| Peritoneal adhesions (postoperative) (postinfection) | digestive | 265995 | 3364 | 0.15 | 0.03 | 1.30E-04 |
| Secondary malignant neoplasm of digestive systems | neoplasms | 238927 | 1763 | 0.20 | 0.04 | 2.35E-04 |
| Anal and rectal conditions | digestive | 270384 | 7753 | 0.09 | 0.02 | 0.001 |
| Aplastic anemia | hematopoietic | 332577 | 12557 | 0.07 | 0.02 | 0.003 |
| Hemorrhoids | circulatory system | 325049 | 21342 | 0.05 | 0.01 | 0.019 |
| Malignant neoplasm of female breast | neoplasms | 199250 | 12056 | 0.06 | 0.02 | 0.025 |
| Arthropathy NOS | musculoskeletal | 338379 | 70810 | 0.03 | 0.01 | 0.029 |
| Ventral hernia | digestive | 297053 | 3345 | 0.11 | 0.03 | 0.029 |
| Multiple sclerosis | neurological | 300462 | 1221 | -0.17 | 0.05 | 0.029 |
| Unspecified monoarthritis | musculoskeletal | 283684 | 16115 | 0.05 | 0.01 | 0.033 |
| Other anemias | hematopoietic | 331736 | 11716 | 0.06 | 0.02 | 0.034 |
| Benign neoplasm of other parts of digestive system | neoplasms | 330088 | 5791 | 0.08 | 0.02 | 0.042 |
| SE: standard error, FDR: false discovery rate | | | | | | |

## Table S8 TreeWAS results when using the polygenic risk score of colorectal cancer (CRC PRS_127_) in the UK Biobank with colorectal cancer cases included

| Meaning | max_b | b_ci_lhs | b_ci_rhs | POST_ACTIVE |
| --- | --- | --- | --- | --- |
| Chapter I Certain infectious and parasitic diseases | 0.04 | 0.04 | 0.05 | 1.00 |
| A30-A49 Other bacterial diseases | 0.04 | 0.04 | 0.05 | 0.95 |
| B95-B98 Bacterial, viral and other infectious agents | 0.04 | 0.04 | 0.05 | 1.00 |
| B95 Streptococcus and staphylococcus as the cause of diseases classified to other chapters | 0.04 | 0.04 | 0.05 | 0.99 |
| B96 Other bacterial agents as the cause of diseases classified to other chapters | 0.04 | 0.04 | 0.05 | 0.97 |
| Chapter II Neoplasms | 0.04 | 0.04 | 0.05 | 1.00 |
| C15-C26 Malignant neoplasms of digestive organs | 0.80 | 0.76 | 0.84 | 1.00 |
| C18 Malignant neoplasm of colon | 0.80 | 0.76 | 0.84 | 1.00 |
| C18.0 Caecum | 0.80 | 0.76 | 0.84 | 1.00 |
| C18.2 Ascending colon | 0.80 | 0.76 | 0.84 | 1.00 |
| C18.3 Hepatic flexure | 0.80 | 0.76 | 0.84 | 1.00 |
| C18.4 Transverse colon | 0.80 | 0.76 | 0.84 | 1.00 |
| C18.5 Splenic flexure | 0.80 | 0.76 | 0.84 | 1.00 |
| C18.6 Descending colon | 0.80 | 0.76 | 0.84 | 1.00 |
| C18.7 Sigmoid colon | 0.80 | 0.76 | 0.84 | 1.00 |
| C18.8 Overlapping lesion of colon | 0.80 | 0.76 | 0.84 | 1.00 |
| C18.9 Colon, unspecified | 0.80 | 0.76 | 0.84 | 1.00 |
| C19 Malignant neoplasm of rectosigmoid junction | 0.80 | 0.76 | 0.84 | 1.00 |
| C20 Malignant neoplasm of rectum | 0.80 | 0.76 | 0.84 | 1.00 |
| C26 Malignant neoplasm of other and ill-defined digestive organs | 0.80 | 0.76 | 0.84 | 1.00 |
| C26.0 Intestinal tract, part unspecified | 0.80 | 0.76 | 0.84 | 1.00 |
| C50-C50 Malignant neoplasm of breast | 0.04 | 0.04 | 0.05 | 1.00 |
| C50 Malignant neoplasm of breast | 0.04 | 0.04 | 0.05 | 0.99 |
| C50.9 Breast, unspecified | 0.04 | 0.04 | 0.05 | 0.98 |
| C60-C63 Malignant neoplasms of male genital organs | 0.04 | 0.04 | 0.05 | 0.97 |
| C76-C80 Malignant neoplasms of ill-defined, secondary and unspecified sites | 0.24 | 0.21 | 0.28 | 1.00 |
| C77 Secondary and unspecified malignant neoplasm of lymph nodes | 0.24 | 0.21 | 0.28 | 1.00 |
| C77.2 Intra-abdominal lymph nodes | 0.24 | 0.21 | 0.29 | 1.00 |
| C77.9 Lymph node, unspecified | 0.24 | 0.21 | 0.28 | 1.00 |
| C78 Secondary malignant neoplasm of respiratory and digestive organs | 0.24 | 0.21 | 0.28 | 1.00 |
| C78.0 Secondary malignant neoplasm of lung | 0.24 | 0.21 | 0.28 | 1.00 |
| C78.6 Secondary malignant neoplasm of retroperitoneum and peritoneum | 0.24 | 0.21 | 0.28 | 1.00 |
| C78.7 Secondary malignant neoplasm of liver | 0.24 | 0.21 | 0.28 | 1.00 |
| D00-D09 In situ neoplasms | 0.04 | 0.04 | 0.05 | 0.97 |
| D01 Carcinoma in situ of other and unspecified digestive organs | 1.02 | 0.81 | 1.23 | 1.00 |
| D01.0 Colon | 1.02 | 0.81 | 1.23 | 1.00 |
| D01.2 Rectum | 1.02 | 0.81 | 1.23 | 1.00 |
| D05 Carcinoma in situ of breast | 0.04 | 0.04 | 0.05 | 0.96 |
| D10-D36 Benign neoplasms | 0.04 | 0.04 | 0.05 | 1.00 |
| D12 Benign neoplasm of colon, rectum, anus and anal canal | 0.68 | 0.66 | 0.70 | 1.00 |
| D12.0 Caecum | 0.68 | 0.66 | 0.70 | 1.00 |
| D12.2 Ascending colon | 0.68 | 0.66 | 0.70 | 1.00 |
| D12.3 Transverse colon | 0.68 | 0.66 | 0.70 | 1.00 |
| D12.4 Descending colon | 0.68 | 0.66 | 0.70 | 1.00 |
| D12.5 Sigmoid colon | 0.68 | 0.66 | 0.70 | 1.00 |
| D12.6 Colon, unspecified | 0.68 | 0.66 | 0.70 | 1.00 |
| D12.7 Rectosigmoid junction | 0.68 | 0.66 | 0.70 | 1.00 |
| D12.8 Rectum | 0.68 | 0.66 | 0.70 | 1.00 |
| D18 Haemangioma and lymphangioma, any site | 0.04 | 0.04 | 0.05 | 0.95 |
| D37 Neoplasm of uncertain or unknown behaviour of oral cavity and digestive organs | 0.04 | 0.04 | 0.98 | 0.91 |
| D37.4 Colon | 0.84 | 0.69 | 0.99 | 1.00 |
| D37.5 Rectum | 0.85 | 0.71 | 1.08 | 1.00 |
| Chapter III Diseases of the blood and blood-forming organs and certain disorders involving the immune mechanism | 0.04 | 0.04 | 0.05 | 1.00 |
| D60-D64 Aplastic and other anaemias | 0.04 | 0.04 | 0.05 | 1.00 |
| D64 Other anaemias | 0.04 | 0.04 | 0.05 | 1.00 |
| D64.9 Anaemia, unspecified | 0.04 | 0.04 | 0.05 | 0.99 |
| D70-D77 Other diseases of blood and blood-forming organs | 0.04 | 0.04 | 0.05 | 0.96 |
| Chapter IV Endocrine, nutritional and metabolic diseases | 0.04 | 0.04 | 0.05 | 0.97 |
| E70-E90 Metabolic disorders | 0.04 | 0.04 | 0.05 | 0.94 |
| E10-E14 Diabetes mellitus | 0.04 | 0.04 | 0.05 | 0.90 |
| Chapter X Diseases of the respiratory system | 0.04 | 0.04 | 0.05 | 1.00 |
| J40-J47 Chronic lower respiratory diseases | 0.04 | 0.04 | 0.05 | 0.98 |
| Chapter XI Diseases of the digestive system | 0.12 | 0.04 | 0.14 | 1.00 |
| K40-K46 Hernia | 0.12 | 0.04 | 0.14 | 0.96 |
| K43 Ventral hernia_int | 0.12 | 0.04 | 0.14 | 0.96 |
| K43.9 Ventral hernia without obstruction or gangrene | 0.12 | 0.04 | 0.14 | 0.95 |
| K55-K64 Other diseases of intestines | 0.12 | 0.11 | 0.14 | 1.00 |
| K56 Paralytic ileus and intestinal obstruction without hernia | 0.12 | 0.11 | 0.14 | 1.00 |
| K56.2 Volvulus | 0.12 | 0.11 | 0.14 | 0.96 |
| K56.5 Intestinal adhesions [bands] with obstruction | 0.12 | 0.11 | 0.14 | 0.98 |
| K56.6 Other and unspecified intestinal obstruction | 0.12 | 0.11 | 0.14 | 1.00 |
| K57 Diverticular disease of intestine | 0.12 | 0.11 | 0.14 | 1.00 |
| K57.3 Diverticular disease of large intestine without perforation or abscess | 0.12 | 0.11 | 0.14 | 1.00 |
| K57.9 Diverticular disease of intestine, part unspecified, without perforation or abscess | 0.12 | 0.11 | 0.14 | 1.00 |
| K62 Other diseases of anus and rectum | 0.12 | 0.11 | 0.14 | 1.00 |
| K62.1 Rectal polyp | 0.44 | 0.40 | 0.48 | 1.00 |
| K62.4 Stenosis of anus and rectum | 0.12 | 0.11 | 0.14 | 0.98 |
| K62.8 Other specified diseases of anus and rectum | 0.12 | 0.11 | 0.14 | 0.95 |
| K62.9 Disease of anus and rectum, unspecified | 0.12 | 0.11 | 0.14 | 0.96 |
| K63 Other diseases of intestine | 0.12 | 0.11 | 0.14 | 1.00 |
| K63.1 Perforation of intestine (nontraumatic) | 0.12 | 0.11 | 0.14 | 0.96 |
| K63.5 Polyp of colon | 0.47 | 0.44 | 0.50 | 1.00 |
| K63.8 Other specified diseases of intestine | 0.12 | 0.11 | 0.14 | 1.00 |
| K63.9 Disease of intestine, unspecified | 0.12 | 0.11 | 0.14 | 1.00 |
| K64 Haemorrhoids and perianal venous thrombosis | 0.12 | 0.11 | 0.14 | 0.97 |
| K65-K67 Diseases of peritoneum | 0.12 | 0.04 | 0.14 | 1.00 |
| K65 Peritonitis | 0.12 | 0.04 | 0.14 | 0.95 |
| K66 Other disorders of peritoneum | 0.12 | 0.04 | 0.14 | 1.00 |
| K66.0 Peritoneal adhesions | 0.12 | 0.04 | 0.14 | 1.00 |
| K90-K93 Other diseases of the digestive system | 0.12 | 0.04 | 0.14 | 0.98 |
| K91 Postprocedural disorders of digestive system, not elsewhere classified | 0.12 | 0.04 | 0.32 | 1.00 |
| K91.3 Postoperative intestinal obstruction | 0.12 | 0.04 | 0.32 | 1.00 |
| K91.4 Colostomy and enterostomy malfunction | 0.12 | 0.04 | 0.32 | 0.98 |
| K91.8 Other postprocedural disorders of digestive system, not elsewhere classified | 0.12 | 0.04 | 0.32 | 0.98 |
| Chapter XII Diseases of the skin and subcutaneous tissue | 0.04 | 0.04 | 0.05 | 1.00 |
| L80-L99 Other disorders of the skin and subcutaneous tissue | 0.04 | 0.04 | 0.05 | 0.98 |
| Chapter XIII Diseases of the musculoskeletal system and connective tissue | 0.04 | 0.04 | 0.05 | 1.00 |
| M15-M19 Arthrosis | 0.04 | 0.04 | 0.05 | 1.00 |
| M17 Gonarthrosis [arthrosis of knee] | 0.04 | 0.04 | 0.05 | 0.99 |
| M17.9 Gonarthrosis, unspecified | 0.04 | 0.04 | 0.05 | 0.99 |
| M20-M25 Other joint disorders | 0.04 | 0.04 | 0.05 | 1.00 |
| M20 Acquired deformities of fingers and toes | 0.04 | 0.04 | 0.05 | 0.98 |
| M20.1 Hallux valgus (acquired) | 0.04 | 0.04 | 0.05 | 0.95 |
| Chapter XIV Diseases of the genitourinary system | 0.04 | 0.04 | 0.05 | 1.00 |
| N17-N19 Renal failure | 0.04 | 0.04 | 0.05 | 0.96 |
| N30-N39 Other diseases of urinary system | 0.04 | 0.04 | 0.05 | 0.99 |
| N39 Other disorders of urinary system | 0.04 | 0.04 | 0.05 | 0.98 |
| N39.0 Urinary tract infection, site not specified | 0.04 | 0.04 | 0.05 | 0.98 |
| max_b: maximum a posteriori effect estimates (beta) and the 95% credible interval (max_b_lhs, max_b_rhs), POST_ACTIVE: posterior probability for the beta (β) estimate in the tree analysis not being zero. | | | | |

## Table S9 Colorectal cancer associated phenotypes identified by Danish Disease Trajectory Browser

| Pre-colorectal cancer diseases | Post-colorectal cancer diseases |
| --- | --- |
| Acute appendicitis | Abscess of anal and rectal regions |
| Acute myocardial infarction | Acute renal failure |
| Angina pectoris | Agranulocytosis |
| Asthma | Anaemia in chronic diseases classified elsewhere |
| Atherosclerosis | Bacterial infection of unspecified site |
| Atrial fibrillation and flutter | Bacterial pneumonia, not elsewhere classified |
| Benign neoplasm of colon, rectum, anus and anal canal | Benign neoplasm of colon, rectum, anus and anal canal |
| Benign neoplasm of ovary | Candidiasis |
| Cardiomyopathy | Chronic kidney disease |
| Cholelithiasis | Cystitis |
| Chronic ischaemic heart disease | Decubitus ulcer and pressure area |
| Diaphragmatic hernia | Delirium, not induced by alcohol and other psychoactive substances |
| Diverticular disease of intestine | Dementia in Alzheimer disease |
| Dorsalgia | Depressive episode |
| Duodenal ulcer | Disorders of mineral metabolism |
| Epilepsy | Erysipelas |
| Essential (primary) hypertension | Fissure and fistula of anal and rectal regions |
| Excessive, frequent and irregular menstruation | Fistulae involving female genital tract |
| Female genital prolapse | Gout |
| Functional dyspepsia | Haemorrhoids and perianal venous thrombosis |
| Gastritis and duodenitis | Hepatic failure, not elsewhere classified |
| Gastro-oesophageal reflux disease | Hypotension |
| Gonarthrosis [arthrosis of knee] | Iron deficiency anaemia |
| Heart failure | Malignant neoplasm of liver and intrahepatic bile ducts |
| Hyperplasia of prostate | Malignant neoplasm of other and ill-defined digestive organs |
| Internal derangement of knee | Malignant neoplasm of other and ill-defined sites |
| Iron deficiency anaemia | Malignant neoplasm of retroperitoneum and peritoneum |
| Leiomyoma of uterus | Malignant neoplasm, without specification of site |
| Malignant neoplasm of prostate | Neuromuscular dysfunction of bladder, not elsewhere classified |
| Mental and behavioural disorders due to use of alcohol | Nutritional marasmus |
| Neoplasm of uncertain or unknown behaviour of oral cavity and digestive organs | Obstructive and reflux uropathy |
| Noninflammatory disorders of ovary, fallopian tube and broad ligament | Osteoporosis with pathological fracture |
| Nonrheumatic aortic valve disorders | Osteoporosis without pathological fracture |
| Nonrheumatic mitral valve disorders | Other abdominal hernia |
| Obesity | Other anaemias |
| Oesophagitis | Other anxiety disorders |
| Other anaemias | Other bacterial intestinal infections |
| Other chronic obstructive pulmonary disease | Other chronic obstructive pulmonary disease |
| Other diseases of anus and rectum | Other diseases of anus and rectum |
| Other disorders of muscle | Other diseases of biliary tract |
| Other intervertebral disc disorders | Other diseases of digestive system |
| Other peripheral vascular diseases | Other diseases of intestine |
| Other polyneuropathies | Other diseases of spinal cord |
| Other rheumatoid arthritis | Other disorders of bladder |
| Other systemic involvement of connective tissue | Other disorders of fluid, electrolyte and acid-base balance |
| Retinal disorders in diseases classified elsewhere | Other disorders of kidney and ureter, not elsewhere classified |
| Seropositive rheumatoid arthritis | Other disorders of penis |
| Spondylosis | Other disorders of peritoneum |
| Type 1 diabetes mellitus | Other disorders of skin and subcutaneous tissue, not elsewhere classified |
| Type 2 diabetes mellitus | Other disorders of urethra |
| Ulcerative colitis | Other disorders of urinary system |
| Unspecified chronic bronchitis | Other endocrine disorders |
| Unspecified diabetes mellitus | Other functional intestinal disorders |
|  | Other noninfective gastroenteritis and colitis |
|  | Other pulmonary heart diseases |
|  | Other sepsis |
|  | Other soft tissue disorders, not elsewhere classified |
|  | Other venous embolism and thrombosis |
|  | Paralytic ileus and intestinal obstruction without hernia |
|  | Peritonitis |
|  | Phlebitis and thrombophlebitis |
|  | Pleural effusion in conditions classified elsewhere |
|  | Pleural effusion, not elsewhere classified |
|  | Pneumonia, organism unspecified |
|  | Pneumonitis due to solids and liquids |
|  | Postprocedural disorders of digestive system, not elsewhere classified |
|  | Postprocedural respiratory disorders, not elsewhere classified |
|  | Pulmonary embolism |
|  | Pulmonary oedema |
|  | Recurrent depressive disorder |
|  | Respiratory failure, not elsewhere classified |
|  | Secondary and unspecified malignant neoplasm of lymph nodes |
|  | Secondary malignant neoplasm of other and unspecified sites |
|  | Secondary malignant neoplasm of respiratory and digestive organs |
|  | Sequelae of malnutrition and other nutritional deficiencies |
|  | Sleep disorders |
|  | Tubulo-interstitial nephritis, not specified as acute or chronic |
|  | Type 2 diabetes mellitus |
|  | Unspecified abdominal hernia |
|  | Unspecified dementia |
|  | Unspecified kidney failure |
|  | Vascular disorders of intestine |
|  | Ventral hernia |
|  | Volume depletion |

## Table S10 Genetic variants for diverticular disease and colorectal cancer risk

| Variant | Chr | Position | Effect allele | Other allele | EAF.DD | Beta.DD | SE.DD | EAF.CRC | Beta.CRC | SE.CRC | Trait | Reference |
| --- | --- | --- | --- | --- | --- | --- | --- | --- | --- | --- | --- | --- |
| rs10049390 | 3 | 133701119 | A | G | 0.75 | 0.001 | 0.001 | 0.72 | 0.058 | 0.014 | CRC | Huyghe et al. |
| rs10152518 | 15 | 68177162 | G | A | 0.18 | -0.001 | 0.001 | 0.19 | 0.074 | 0.016 | CRC | Law et al. |
| rs10161980 | 13 | 34093518 | C | G | 0.61 | 3.22E-04 | 0.001 | 0.62 | 0.071 | 0.013 | CRC | Law et al. |
| rs1078643 | 17 | 10707241 | A | G | 0.76 | 0.001 | 0.001 | 0.76 | 0.068 | 0.015 | CRC | Huyghe et al. |
| rs10821907 | 10 | 52648454 | C | T | 0.82 | 0.001 | 0.001 | 0.83 | 0.077 | 0.016 | CRC | Huyghe et al. |
| rs10849432 | 12 | 6385727 | T | C | 0.89 | 0.002 | 0.001 | 0.90 | 0.065 | 0.021 | CRC | Law et al. |
| rs10849438 | 12 | 6412036 | G | T | 0.12 | 0.001 | 0.001 | 0.12 | 0.107 | 0.026 | CRC | Law et al. |
| rs10951878 | 7 | 46926695 | C | T | 0.49 | 4.71E-04 | 0.001 | 0.49 | 0.051 | 0.012 | CRC | Law et al. |
| rs10980628 | 9 | 113671403 | C | T | 0.21 | 1.17E-04 | 0.001 | 0.21 | 0.049 | 0.013 | CRC | Huyghe et al. |
| rs11087784 | 20 | 7740976 | G | A | 0.14 | 0.001 | 0.001 | 0.15 | 0.086 | 0.016 | CRC | Huyghe et al. |
| rs11196170 | 10 | 114722621 | A | G | 0.21 | -3.40E-04 | 0.001 | 0.22 | 0.058 | 0.014 | CRC | Huyghe et al. |
| rs11610543 | 12 | 43134191 | G | A | 0.48 | 0.001 | 0.001 | 0.51 | 0.049 | 0.011 | CRC | Huyghe et al. |
| rs11692435 | 2 | 98275354 | G | A | 0.92 | -2.01E-04 | 0.001 | 0.90 | 0.105 | 0.028 | CRC | Law et al. |
| rs11874392 | 18 | 46453156 | A | T | 0.54 | 0.002 | 0.001 | 0.55 | 0.148 | 0.011 | CRC | Huyghe et al. |
| rs11884596 | 2 | 199612407 | C | T | 0.38 | 0.001 | 0.001 | 0.39 | 0.049 | 0.014 | CRC | Huyghe et al. |
| rs12144319 | 1 | 55246035 | C | T | 0.24 | 0.002 | 0.001 | 0.27 | 0.068 | 0.014 | CRC | Huyghe et al. |
| rs12149163 | 16 | 86339315 | T | C | 0.49 | 0.001 | 0.001 | 0.50 | 0.058 | 0.012 | CRC | Huyghe et al. |
| rs12246635 | 10 | 114288619 | C | T | 0.10 | 0.002 | 0.001 | 0.10 | 0.077 | 0.020 | CRC | Huyghe et al. |
| rs12372718 | 12 | 51171090 | G | A | 0.41 | 0.002 | 0.001 | 0.39 | 0.086 | 0.012 | CRC | Huyghe et al. |
| rs12514517 | 5 | 40280076 | A | G | 0.29 | -4.02E-04 | 0.001 | 0.29 | 0.086 | 0.013 | CRC | Huyghe et al. |
| rs12522693 | 5 | 130195731 | G | A | 0.85 | 1.57E-04 | 0.001 | 0.85 | -0.014 | 0.019 | CRC | Law et al. |
| rs12603526 | 17 | 800593 | C | T | 0.02 | 0.002 | 0.002 | 0.02 | 0.004 | 0.041 | CRC | Law et al. |
| rs12635946 | 3 | 112916918 | C | T | 0.62 | 0.001 | 0.001 | 0.62 | 0.089 | 0.013 | CRC | Law et al. |
| rs12672022 | 7 | 45136423 | T | C | 0.82 | 1.64E-04 | 0.001 | 0.84 | 0.068 | 0.015 | CRC | Huyghe et al. |
| rs12979278 | 19 | 49218602 | T | C | 0.55 | 0.001 | 0.001 | 0.53 | 0.075 | 0.015 | CRC | Law et al. |
| rs13020391 | 2 | 219184436 | C | T | 0.62 | 4.95E-04 | 0.001 | 0.63 | 0.082 | 0.013 | CRC | Law et al. |
| rs13149359 | 4 | 94938618 | A | C | 0.38 | 0.001 | 0.001 | 0.37 | 0.058 | 0.013 | CRC | Huyghe et al. |
| rs1321310 | 6 | 36623124 | C | T | 0.24 | -0.001 | 0.001 | 0.24 | 0.088 | 0.014 | CRC | Law et al. |
| rs1391441 | 4 | 106128760 | A | G | 0.69 | 0.001 | 0.001 | 0.66 | 0.049 | 0.013 | CRC | Huyghe et al. |
| rs1412834 | 9 | 22110131 | T | C | 0.49 | 0.001 | 0.001 | 0.50 | 0.096 | 0.012 | CRC | Law et al. |
| rs143635270 | 16 | 9242047 | G | A | 0.99 | 0.003 | 0.002 | 0.99 | 0.077 | 0.061 | CRC | Huyghe et al. |
| rs16878812 | 6 | 35569562 | A | G | 0.88 | 0.002 | 0.001 | 0.89 | 0.105 | 0.020 | CRC | Law et al. |
| rs16892766 | 8 | 117630683 | C | A | 0.08 | 0.001 | 0.001 | 0.09 | 0.231 | 0.021 | CRC | Law et al. |
| rs16969681 | 15 | 32993111 | T | C | 0.08 | 0.002 | 0.001 | 0.09 | 0.192 | 0.031 | CRC | Law et al. |
| rs17011141 | 1 | 222112634 | G | A | 0.20 | -0.001 | 0.001 | 0.21 | 0.086 | 0.014 | CRC | Huyghe et al. |
| rs17035289 | 4 | 106048291 | T | C | 0.84 | 0.002 | 0.001 | 0.83 | 0.101 | 0.016 | CRC | Law et al. |
| rs17094983 | 14 | 59189361 | G | A | 0.88 | 0.001 | 0.001 | 0.88 | 0.095 | 0.017 | CRC | Huyghe et al. |
| rs1741640 | 20 | 60932414 | C | T | 0.76 | 4.59E-04 | 0.001 | 0.77 | 0.165 | 0.016 | CRC | Law et al. |
| rs174533 | 11 | 61549025 | G | A | 0.65 | 2.59E-04 | 0.001 | 0.67 | 0.077 | 0.012 | CRC | Huyghe et al. |
| rs17816465 | 15 | 33156386 | A | G | 0.20 | 0.001 | 0.001 | 0.20 | 0.092 | 0.023 | CRC | Law et al. |
| rs17836917 | 17 | 32047282 | G | A | 0.98 | -0.001 | 0.002 | 0.98 | 0.057 | 0.071 | CRC | Law et al. |
| rs189583 | 20 | 6376457 | G | C | 0.33 | 0.001 | 0.001 | 0.33 | 0.095 | 0.013 | CRC | Huyghe et al. |
| rs2070699 | 6 | 12292772 | T | G | 0.47 | -1.94E-04 | 0.001 | 0.48 | 0.056 | 0.017 | CRC | Law et al. |
| rs2179593 | 20 | 42660286 | A | C | 0.72 | -2.39E-04 | 0.001 | 0.72 | 0.063 | 0.014 | CRC | Law et al. |
| rs2186607 | 11 | 101656397 | T | A | 0.51 | 1.49E-04 | 0.001 | 0.51 | 0.049 | 0.011 | CRC | Huyghe et al. |
| rs2193352 | 10 | 101346609 | G | A | 0.19 | 0.002 | 0.001 | 0.19 | 0.112 | 0.015 | CRC | Law et al. |
| rs2250430 | 12 | 6421174 | T | A | 0.71 | 0.001 | 0.001 | 0.71 | 0.068 | 0.014 | CRC | Huyghe et al. |
| rs2295444 | 20 | 33173883 | C | T | 0.51 | -7.90E-05 | 0.001 | 0.50 | 0.020 | 0.012 | CRC | Law et al. |
| rs2516420 | 6 | 31449620 | C | T | 0.92 | -0.002 | 0.001 | 0.93 | 0.058 | 0.023 | CRC | Huyghe et al. |
| rs2735940 | 5 | 1296486 | G | A | 0.49 | -0.001 | 0.001 | 0.50 | 0.095 | 0.012 | CRC | Huyghe et al. |
| rs28488 | 20 | 6762221 | T | C | 0.62 | 0.001 | 0.001 | 0.65 | 0.077 | 0.013 | CRC | Huyghe et al. |
| rs28840750 | 19 | 33519927 | T | G | 0.96 | 0.001 | 0.001 | 0.95 | 0.199 | 0.025 | CRC | Huyghe et al. |
| rs3087967 | 11 | 111156836 | T | C | 0.30 | 0.001 | 0.001 | 0.30 | 0.146 | 0.017 | CRC | Law et al. |
| rs3125049 | 6 | 160754712 | G | A | 0.84 | -3.49E-04 | 0.001 | 0.85 | 0.020 | 0.022 | CRC | Huyghe et al. |
| rs3131043 | 6 | 30758466 | G | A | 0.42 | -2.24E-04 | 0.001 | 0.43 | 0.050 | 0.028 | CRC | Law et al. |
| rs3133285 | 8 | 117629411 | G | C | 0.83 | 0.001 | 0.001 | 0.82 | 0.068 | 0.015 | CRC | Huyghe et al. |
| rs3217810 | 12 | 4388271 | T | C | 0.13 | 0.001 | 0.001 | 0.13 | 0.148 | 0.020 | CRC | Huyghe et al. |
| rs3217874 | 12 | 4400808 | T | C | 0.44 | -0.001 | 0.001 | 0.43 | 0.049 | 0.013 | CRC | Huyghe et al. |
| rs34405347 | 9 | 101679752 | T | G | 0.91 | 0.001 | 0.001 | 0.90 | 0.077 | 0.019 | CRC | Huyghe et al. |
| rs34797592 | 19 | 16417198 | T | C | 0.11 | 0.001 | 0.001 | 0.12 | 0.086 | 0.019 | CRC | Huyghe et al. |
| rs35107139 | 14 | 54419106 | C | A | 0.40 | 0.001 | 0.001 | 0.42 | 0.086 | 0.012 | CRC | Huyghe et al. |
| rs35446936 | 3 | 169486508 | G | A | 0.76 | 0.001 | 0.001 | 0.70 | 0.073 | 0.019 | CRC | Law et al. |
| rs35470271 | 3 | 40915239 | G | A | 0.15 | 0.002 | 0.001 | 0.15 | 0.122 | 0.015 | CRC | Huyghe et al. |
| rs35808169 | 12 | 4368607 | C | T | 0.16 | 0.001 | 0.001 | 0.17 | 0.095 | 0.015 | CRC | Huyghe et al. |
| rs3787089 | 20 | 62316630 | C | T | 0.33 | 0.003 | 0.001 | 0.32 | 0.067 | 0.014 | CRC | Law et al. |
| rs3801081 | 7 | 47511161 | G | A | 0.66 | 0.001 | 0.001 | 0.68 | 0.071 | 0.021 | CRC | Law et al. |
| rs3987 | 4 | 118759055 | G | A | 0.37 | 2.75E-04 | 0.001 | 0.38 | 0.012 | 0.013 | CRC | Law et al. |
| rs4313119 | 8 | 128571855 | G | T | 0.76 | -0.001 | 0.001 | 0.75 | 0.077 | 0.014 | CRC | Huyghe et al. |
| rs4450168 | 11 | 10286755 | C | A | 0.18 | 0.001 | 0.001 | 0.17 | 0.094 | 0.019 | CRC | Law et al. |
| rs448513 | 2 | 159964552 | C | T | 0.32 | 1.81E-04 | 0.001 | 0.34 | 0.058 | 0.013 | CRC | Huyghe et al. |
| rs45597035 | 13 | 73649152 | A | G | 0.65 | -0.001 | 0.001 | 0.64 | 0.074 | 0.013 | CRC | Law et al. |
| rs4759277 | 12 | 57533690 | A | C | 0.36 | 0.001 | 0.001 | 0.34 | 0.068 | 0.014 | CRC | Huyghe et al. |
| rs4776316 | 15 | 67007813 | A | G | 0.72 | 0.001 | 0.001 | 0.73 | 0.078 | 0.019 | CRC | Law et al. |
| rs4811050 | 20 | 48980670 | A | G | 0.18 | 0.002 | 0.001 | 0.18 | 0.101 | 0.019 | CRC | Law et al. |
| rs4917993 | 10 | 104791595 | C | G | 0.87 | -0.001 | 0.001 | 0.87 | 0.068 | 0.018 | CRC | Huyghe et al. |
| rs4944940 | 11 | 74415252 | G | A | 0.96 | 0.004 | 0.001 | 0.96 | 0.269 | 0.037 | CRC | Law et al. |
| rs4968127 | 17 | 809643 | G | A | 0.36 | 0.001 | 0.001 | 0.37 | 0.058 | 0.012 | CRC | Huyghe et al. |
| rs4976270 | 5 | 134467220 | C | T | 0.54 | -0.001 | 0.001 | 0.55 | 0.068 | 0.011 | CRC | Huyghe et al. |
| rs55990915 | 12 | 117763309 | A | C | 0.14 | 2.81E-04 | 0.001 | 0.13 | 0.095 | 0.019 | CRC | Huyghe et al. |
| rs56324967 | 15 | 67402824 | C | T | 0.68 | -0.001 | 0.001 | 0.66 | 0.068 | 0.013 | CRC | Huyghe et al. |
| rs58658771 | 15 | 33001734 | A | T | 0.18 | 0.001 | 0.001 | 0.19 | 0.122 | 0.015 | CRC | Huyghe et al. |
| rs597808 | 12 | 111973358 | G | A | 0.52 | 0.001 | 0.001 | 0.52 | 0.086 | 0.013 | CRC | Huyghe et al. |
| rs6063514 | 20 | 49055318 | C | T | 0.61 | 0.001 | 0.001 | 0.61 | 0.068 | 0.013 | CRC | Huyghe et al. |
| rs6065668 | 20 | 42532821 | C | T | 0.73 | -0.001 | 0.001 | 0.74 | -0.014 | 0.014 | CRC | Law et al. |
| rs6066825 | 20 | 47340117 | A | G | 0.64 | 0.001 | 0.001 | 0.65 | 0.089 | 0.013 | CRC | Law et al. |
| rs6085661 | 20 | 6693128 | T | C | 0.38 | 0.001 | 0.001 | 0.39 | 0.088 | 0.012 | CRC | Law et al. |
| rs6091213 | 20 | 49384745 | C | T | 0.26 | 0.001 | 0.001 | 0.26 | 0.077 | 0.016 | CRC | Law et al. |
| rs61336918 | 16 | 80007266 | A | T | 0.29 | 0.001 | 0.001 | 0.29 | 0.094 | 0.014 | CRC | Law et al. |
| rs61776719 | 1 | 38461319 | C | A | 0.44 | 0.001 | 0.001 | 0.45 | 0.070 | 0.014 | CRC | Law et al. |
| rs62396735 | 6 | 41702582 | C | T | 0.28 | 0.001 | 0.001 | 0.29 | 0.030 | 0.014 | CRC | Huyghe et al. |
| rs62404966 | 6 | 55712124 | C | T | 0.75 | 0.001 | 0.001 | 0.76 | 0.058 | 0.013 | CRC | Huyghe et al. |
| rs6678517 | 1 | 183002639 | A | G | 0.58 | 3.16E-04 | 0.001 | 0.59 | 0.049 | 0.012 | CRC | Huyghe et al. |
| rs6781752 | 3 | 66365163 | A | G | 0.20 | 0.001 | 0.001 | 0.21 | 0.068 | 0.015 | CRC | Huyghe et al. |
| rs6928864 | 6 | 105966894 | C | A | 0.91 | 0.001 | 0.001 | 0.91 | 0.126 | 0.022 | CRC | Law et al. |
| rs6933790 | 6 | 41672769 | T | C | 0.83 | 0.002 | 0.001 | 0.83 | 0.085 | 0.021 | CRC | Law et al. |
| rs6983267 | 8 | 128413305 | G | T | 0.52 | 0.001 | 0.001 | 0.52 | 0.140 | 0.012 | CRC | Huyghe et al. |
| rs704017 | 10 | 80819132 | G | A | 0.57 | -6.18E-05 | 0.001 | 0.58 | 0.077 | 0.012 | CRC | Huyghe et al. |
| rs7121958 | 11 | 74280012 | G | T | 0.50 | 0.002 | 0.001 | 0.51 | 0.086 | 0.012 | CRC | Huyghe et al. |
| rs7160450 | 14 | 54457231 | T | C | 0.38 | 0.001 | 0.001 | 0.38 | 0.049 | 0.013 | CRC | Huyghe et al. |
| rs72647484 | 1 | 22587728 | T | C | 0.91 | -0.001 | 0.001 | 0.91 | 0.122 | 0.030 | CRC | Law et al. |
| rs72942485 | 3 | 112999560 | G | A | 0.98 | -3.01E-04 | 0.002 | 0.98 | 0.148 | 0.040 | CRC | Huyghe et al. |
| rs7300312 | 12 | 115890922 | C | T | 0.58 | 0.001 | 0.001 | 0.57 | 0.068 | 0.012 | CRC | Huyghe et al. |
| rs73068325 | 19 | 59079096 | T | C | 0.18 | -0.002 | 0.001 | 0.18 | 0.058 | 0.016 | CRC | Huyghe et al. |
| rs7333607 | 13 | 37462010 | G | A | 0.24 | 4.97E-04 | 0.001 | 0.23 | 0.086 | 0.015 | CRC | Huyghe et al. |
| rs73975588 | 17 | 816741 | A | C | 0.87 | 3.62E-04 | 0.001 | 0.87 | 0.103 | 0.026 | CRC | Law et al. |
| rs7398375 | 12 | 57540848 | C | G | 0.73 | 0.002 | 0.001 | 0.72 | 0.089 | 0.017 | CRC | Law et al. |
| rs7495132 | 15 | 91172901 | T | C | 0.12 | -2.97E-05 | 0.001 | 0.12 | 0.112 | 0.025 | CRC | Law et al. |
| rs75610640 | 2 | 192562764 | C | T | 0.16 | 3.86E-04 | 0.001 | 0.16 | 0.049 | 0.017 | CRC | Huyghe et al. |
| rs75686861 | 4 | 145621328 | A | G | 0.09 | -0.004 | 0.001 | 0.10 | 0.118 | 0.021 | CRC | Law et al. |
| rs75954926 | 17 | 81061048 | G | A | 0.66 | 5.18E-05 | 0.001 | 0.65 | 0.095 | 0.014 | CRC | Huyghe et al. |
| rs7708610 | 5 | 40102443 | A | G | 0.36 | 0.001 | 0.001 | 0.35 | 0.049 | 0.013 | CRC | Huyghe et al. |
| rs77776598 | 5 | 1240998 | C | T | 0.05 | -3.31E-04 | 0.001 | 0.06 | 0.144 | 0.027 | CRC | Law et al. |
| rs78341008 | 13 | 73791554 | C | T | 0.07 | 4.25E-04 | 0.001 | 0.07 | 0.122 | 0.025 | CRC | Huyghe et al. |
| rs7993934 | 13 | 111074915 | T | C | 0.64 | 3.31E-04 | 0.001 | 0.65 | 0.070 | 0.013 | CRC | Law et al. |
| rs812481 | 3 | 66442435 | G | C | 0.52 | 0.001 | 0.001 | 0.52 | 0.006 | 0.012 | CRC | Law et al. |
| rs847208 | 16 | 86254051 | A | C | 0.63 | 0.001 | 0.001 | 0.63 | 0.014 | 0.018 | CRC | Law et al. |
| rs899244 | 16 | 86700030 | T | C | 0.21 | 0.003 | 0.001 | 0.21 | 0.089 | 0.018 | CRC | Law et al. |
| rs9271695 | 6 | 32593080 | G | A | 0.82 | 0.001 | 0.001 | 0.80 | 0.086 | 0.016 | CRC | Huyghe et al. |
| rs9797885 | 19 | 41873001 | G | A | 0.71 | 2.52E-04 | 0.001 | 0.71 | 0.072 | 0.013 | CRC | Law et al. |
| rs9831861 | 3 | 53088285 | G | T | 0.58 | 0.001 | 0.001 | 0.59 | 0.061 | 0.012 | CRC | Law et al. |
| rs983318 | 17 | 70413253 | A | G | 0.24 | -3.91E-04 | 0.001 | 0.25 | 0.068 | 0.014 | CRC | Huyghe et al. |
| rs983402 | 2 | 199781586 | T | C | 0.33 | 0.001 | 0.001 | 0.32 | 0.049 | 0.012 | CRC | Huyghe et al. |
| rs9924886 | 16 | 68743939 | A | C | 0.73 | 0.001 | 0.001 | 0.73 | 0.058 | 0.014 | CRC | Huyghe et al. |
| rs994308 | 20 | 6603622 | C | T | 0.60 | 2.90E-04 | 0.001 | 0.58 | 0.049 | 0.012 | CRC | Huyghe et al. |
| rs1330889 | 13 | 78609615 | C | T | 0.88 | -3.53E-04 | 0.001 | 0.87 | 0.101 | 0.016 | CRC | Law et al. |
| rs186722897 | 4 | 163374639 | T | A | 0.04 | 0.001 | 0.002 | 0.05 | 0.093 | 0.030 | CRC | Law et al. |
| rs10173528 | 2 | 28065525 | T | C | 0.60 | -0.003 | 0.001 | 0.59 | -0.021 | 0.017 | DD | Maguire 2018 |
| rs10472291 | 5 | 37772678 | A | C | 0.35 | 0.004 | 0.001 | 0.34 | -0.017 | 0.013 | DD | Maguire 2018 |
| rs115490395 | 1 | 110120397 | A | C | 0.01 | 0.016 | 0.003 | 0.01 | -0.006 | 0.086 | DD | Maguire 2018 |
| rs11934833 | 4 | 156636431 | G | C | 0.29 | -0.003 | 0.001 | 0.30 | -0.017 | 0.013 | DD | Maguire 2018 |
| rs12293535 | 11 | 14993308 | A | G | 0.29 | 0.004 | 0.001 | 0.27 | 0.022 | 0.015 | DD | Maguire 2018 |
| rs1381335 | 8 | 119415408 | T | C | 0.23 | -0.004 | 0.001 | 0.23 | 0.002 | 0.016 | DD | Maguire 2018 |
| rs2049865 | 8 | 115576319 | A | C | 0.59 | 0.003 | 0.001 | 0.58 | -4.32E-04 | 0.013 | DD | Maguire 2018 |
| rs2784255 | 1 | 220893031 | C | T | 0.47 | -0.003 | 0.001 | 0.49 | 0.024 | 0.017 | DD | Maguire 2018 |
| rs6949391 | 7 | 102806416 | T | C | 0.35 | 0.004 | 0.001 | 0.34 | -0.009 | 0.022 | DD | Maguire 2018 |
| rs72945112 | 11 | 70247466 | T | C | 0.14 | -0.003 | 0.001 | 0.14 | 0.067 | 0.052 | DD | Maguire 2018 |
| rs75434097 | 21 | 45999606 | A | G | 0.15 | 0.005 | 0.001 | 0.14 | -0.001 | 0.019 | DD | Maguire 2018 |
| rs8074740 | 17 | 44235410 | A | G | 0.33 | 0.004 | 0.001 | 0.32 | -0.010 | 0.014 | DD | Maguire 2018 |
| rs875107 | 11 | 70159268 | A | C | 0.50 | -0.003 | 0.001 | 0.53 | -0.016 | 0.013 | DD | Maguire 2018 |
| rs9856118 | 3 | 151360428 | G | A | 0.16 | -0.005 | 0.001 | 0.17 | 0.001 | 0.016 | DD | Maguire 2018 |
| rs10471645 | 5 | 64295363 | T | C | 0.17 | 0.005 | 0.001 | 0.17 | 0.043 | 0.026 | DD | Schafmayer 2019 |
| rs111316530 | 21 | 47399453 | T | TGTGTGTCA | 0.15 | 0.005 | 0.001 | 0.15 | -0.009 | 0.018 | DD | Schafmayer 2019 |
| rs112609918 | 7 | 73427600 | A | T | 0.95 | -0.009 | 0.001 | 0.95 | -0.040 | 0.035 | DD | Schafmayer 2019 |
| rs12041565 | 1 | 245773041 | C | T | 0.82 | -0.004 | 0.001 | 0.82 | -0.023 | 0.017 | DD | Schafmayer 2019 |
| rs12942267 | 17 | 7372637 | C | T | 0.36 | -0.004 | 0.001 | 0.37 | 0.021 | 0.013 | DD | Schafmayer 2019 |
| rs139760870 | 10 | 124168942 | TTATAGTGGAAATATA | T | 0.94 | 0.006 | 0.001 | 0.94 | -0.013 | 0.026 | DD | Schafmayer 2019 |
| rs1473813 | 13 | 33727605 | G | A | 0.61 | 0.003 | 0.001 | 0.61 | -0.011 | 0.013 | DD | Schafmayer 2019 |
| rs147496465 | 9 | 78739440 | A | ATCAGGAGG | 0.47 | -0.003 | 0.001 | 0.48 | -0.001 | 0.019 | DD | Schafmayer 2019 |
| rs17309930 | 11 | 27748493 | C | A | 0.79 | 0.006 | 0.001 | 0.80 | 0.024 | 0.015 | DD | Schafmayer 2019 |
| rs1802575 | 2 | 56093204 | G | C | 0.87 | -0.007 | 0.001 | 0.87 | 0.003 | 0.018 | DD | Schafmayer 2019 |
| rs1888693 | 10 | 18440444 | G | A | 0.66 | 0.004 | 0.001 | 0.65 | -0.048 | 0.013 | DD | Schafmayer 2019 |
| rs1973232 | 17 | 76856966 | G | A | 0.19 | 0.004 | 0.001 | 0.18 | 0.013 | 0.016 | DD | Schafmayer 2019 |
| rs2056544 | 15 | 76826003 | G | A | 0.42 | -0.004 | 0.001 | 0.43 | 0.012 | 0.012 | DD | Schafmayer 2019 |
| rs208814 | 20 | 37493576 | A | G | 0.36 | 0.003 | 0.001 | 0.35 | -0.007 | 0.018 | DD | Schafmayer 2019 |
| rs2131755 | 16 | 84857378 | A | G | 0.59 | -0.004 | 0.001 | 0.59 | 0.010 | 0.013 | DD | Schafmayer 2019 |
| rs2280028 | 16 | 86233413 | G | A | 0.86 | 0.005 | 0.001 | 0.87 | 0.034 | 0.024 | DD | Schafmayer 2019 |
| rs3113037 | 7 | 96078564 | C | T | 0.77 | -0.004 | 0.001 | 0.76 | 0.002 | 0.014 | DD | Schafmayer 2019 |
| rs34126945 | 5 | 122329729 | A | G | 0.67 | 0.003 | 0.001 | 0.68 | 0.007 | 0.013 | DD | Schafmayer 2019 |
| rs3732760 | 3 | 151074941 | A | C | 0.63 | -0.004 | 0.001 | 0.62 | -0.002 | 0.014 | DD | Schafmayer 2019 |
| rs3752946 | 10 | 105670849 | A | T | 0.45 | 0.003 | 0.001 | 0.44 | 0.048 | 0.013 | DD | Schafmayer 2019 |
| rs3775010 | 4 | 95821419 | T | C | 0.64 | -0.004 | 0.001 | 0.65 | 0.014 | 0.013 | DD | Schafmayer 2019 |
| rs387505 | 15 | 68238462 | T | C | 0.44 | 0.003 | 0.001 | 0.44 | 0.007 | 0.012 | DD | Schafmayer 2019 |
| rs4132788 | 4 | 15386383 | C | T | 0.74 | -0.003 | 0.001 | 0.73 | 0.013 | 0.014 | DD | Schafmayer 2019 |
| rs4333882 | 1 | 234352899 | A | G | 0.81 | -0.007 | 0.001 | 0.79 | 0.043 | 0.020 | DD | Schafmayer 2019 |
| rs4802297 | 19 | 38738130 | G | C | 0.48 | 0.004 | 0.001 | 0.49 | 0.007 | 0.016 | DD | Schafmayer 2019 |
| rs4871180 | 8 | 122259074 | C | T | 0.75 | -0.004 | 0.001 | 0.74 | 0.036 | 0.014 | DD | Schafmayer 2019 |
| rs505922 | 9 | 136149229 | T | C | 0.68 | 0.005 | 0.001 | 0.67 | -0.008 | 0.013 | DD | Schafmayer 2019 |
| rs6001870 | 22 | 40695172 | A | C | 0.66 | -0.003 | 0.001 | 0.75 | 0.005 | 0.023 | DD | Schafmayer 2019 |
| rs61814883 | 1 | 151970629 | G | A | 0.70 | 0.004 | 0.001 | 0.70 | 0.040 | 0.015 | DD | Schafmayer 2019 |
| rs61823192 | 1 | 219294570 | C | T | 0.97 | 0.013 | 0.002 | 0.98 | 0.231 | 0.170 | DD | Schafmayer 2019 |
| rs62125298 | 2 | 18937283 | G | T | 0.83 | 0.004 | 0.001 | 0.84 | -0.001 | 0.018 | DD | Schafmayer 2019 |
| rs6714546 | 2 | 33361425 | A | G | 0.29 | 0.003 | 0.001 | 0.28 | 0.013 | 0.014 | DD | Schafmayer 2019 |
| rs6734367 | 2 | 144314247 | T | G | 0.18 | 0.011 | 0.001 | 0.17 | -0.027 | 0.025 | DD | Schafmayer 2019 |
| rs7077800 | 10 | 25819228 | C | T | 0.53 | 0.005 | 0.001 | 0.53 | -0.031 | 0.012 | DD | Schafmayer 2019 |
| rs7098322 | 10 | 101391169 | C | T | 0.13 | 0.006 | 0.001 | 0.12 | -0.034 | 0.023 | DD | Schafmayer 2019 |
| rs71472433 | 15 | 40649609 | A | C | 0.83 | -0.005 | 0.001 | 0.84 | 0.034 | 0.017 | DD | Schafmayer 2019 |
| rs7609897 | 3 | 15502681 | G | T | 0.79 | 0.007 | 0.001 | 0.78 | -0.022 | 0.016 | DD | Schafmayer 2019 |
| rs7624168 | 3 | 5843836 | A | G | 0.22 | -0.004 | 0.001 | 0.46 | 0.025 | 0.013 | DD | Schafmayer 2019 |
| rs7990 | 6 | 32609965 | C | A | 0.87 | -0.005 | 0.001 | 0.90 | -0.077 | 0.048 | DD | Schafmayer 2019 |
| rs9482094 | 6 | 98364895 | A | G | 0.37 | -0.004 | 0.001 | 0.36 | -0.005 | 0.013 | DD | Schafmayer 2019 |
| rs9520339 | 13 | 107897823 | C | T | 0.76 | 0.005 | 0.001 | 0.76 | -0.013 | 0.017 | DD | Schafmayer 2019 |
| rs9555371 | 13 | 108215404 | A | G | 0.80 | 0.005 | 0.001 | 0.80 | -0.005 | 0.017 | DD | Schafmayer 2019 |
| rs9960286 | 18 | 20028737 | A | G | 0.76 | -0.004 | 0.001 | 0.76 | 0.008 | 0.019 | DD | Schafmayer 2019 |
| EAF: effect allele frequency, DD: diverticular disease, CRC colorectal cancer, SE: standard error | | | | | | | | | | | | |

## Table S11 Results of genetic correlation and bi-directional Mendelian randomisation analyses for the association between CRC and diverticular disease.

| Genetic correlation | | Bi-direction Mendelian randomisation | | | | |
| --- | --- | --- | --- | --- | --- | --- |
|  |  |  | Diverticular disease on CRC | | CRC on diverticular disease | |
| Correlation coefficient (95%CI) | P | Methods | OR (95%CI) | P | OR (95%CI) | P |
| 0.07 (-0.07, 0.21) | 0.33 | IVW | 0.37 (0.10, 1.36) | 0.14 | 1.008 (1.006, 1.010) | 9.78×10^-18^ |
|  |  | MR-Egger | 0.04 (0.0004, 4.89) | 0.19 | 1.006 (1.001, 1.010) | 0.013 |
|  |  | MR-Egger-intercept | / | 0.36 | / | 0.27 |
| OR: odds ratio, CI: confidence interval, CRC: colorectal cancer, IVW: inverse variance weighted | | | | | | |

# Supplementary Figures

## Figure S1 The process of colorectal cancer risk SNPs selection for generating the polygenic risk score

SNP: single nucleotide polymorphism, CRC: colorectal cancer, LD: linkage disequilibrium,


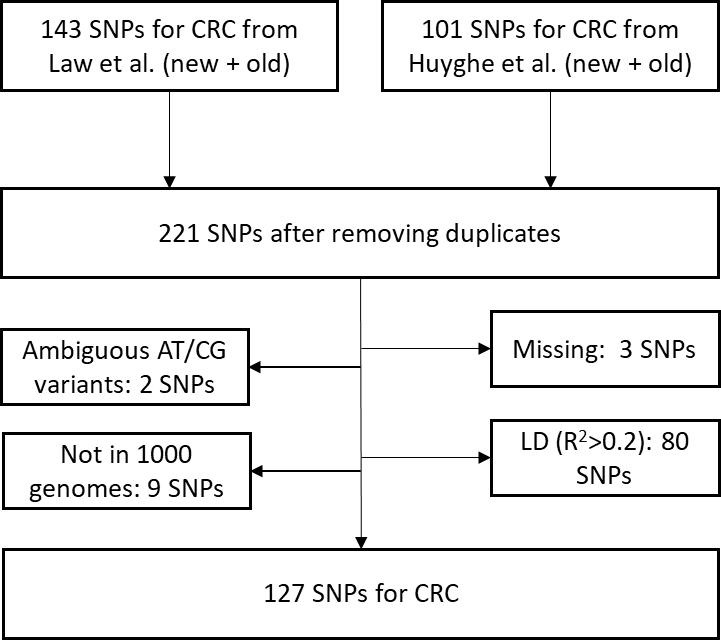


## Figure S2 Sample quality control in the UK Biobank


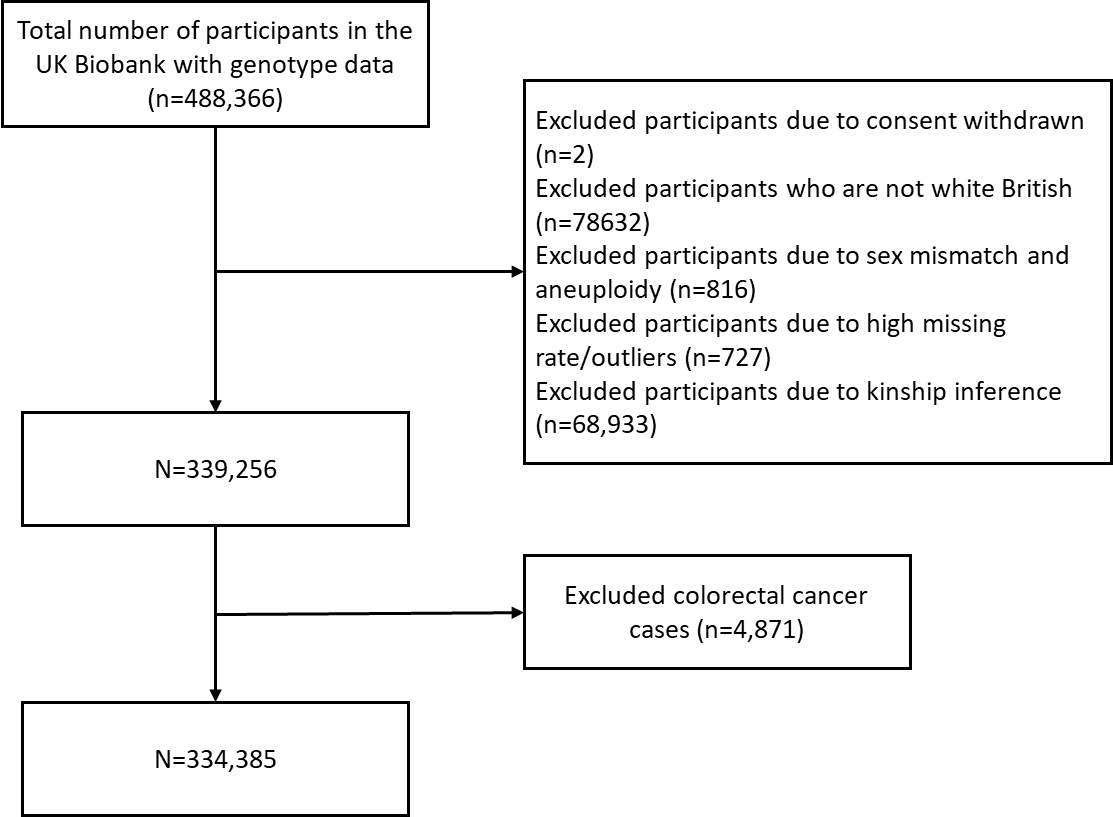


## Figure S3 Proportion of cases in 10 equal deciles of colorectal cancer polygenic risk score (CRC PRS_127_) in UK Biobank with or without colorectal cancer cases

The horizontal axis is the 10 equal deciles of colorectal cancer (CRC) polygenic risk score (PRS), and the vertical axis is the proportion of cases in each decile (for A, the vertical axis is the proportion of participants in each decile). The blue bars are the case proportions among the 339,256 participants with CRC cases and the grey bars are the case proportions among 334,385 participants after removing CRC cases. P is the result of paired t-test by comparing the difference of the case proportions in the highest and lowest PRS decile between the datasets with and without CRC cases.

A: the proportion of participants in each CRC PRS decile between the datasets with and without CRC cases.

B: the proportion of cases of type 2 diabetes (ICD10: E11), other anaemia (ICD10: D64), renal failure (ICD10: N17-N19), bacterial infection (ICD10: B95 and B96), gonarthrosis (ICD10: M17), and secondary malignancies in lymph nodes (ICD10: C77), lungs (ICD10: C78.0), peritoneum (ICD10: C78.6), or liver (ICD10: C78.7) in each CRC PRS decile between the datasets with and without CRC cases. These phenotypes were selected based on two criteria: 1) the PheWAS or TreeWAS detected their association with CRC PRS, but the association disappeared after removing CRC cases from the dataset; 2) by searching Danish Disease Trajectory Browser, these phenotypes were associated with CRC in the observational setting.

C: the proportion of cases of benign neoplasm of colon, rectum, anus and anal canal (ICD10: D12), colon polyp (ICD10: K63.5), rectal polyp (ICD10: K62.1), and diverticular disease (ICD10: K57) in each CRC PRS decile between the datasets with and without CRC cases. These phenotypes were associated with CRC PRS in our main analysis. We presented these phenotypes as comparisons with phenotypes in B.

# Reference

1 Bycroft C, Freeman C, Petkova D, Band G, Elliott LT, Sharp K, et al. The UK Biobank resource with deep phenotyping and genomic data. Nature. 2018;562:203-209.

2 McCarthy S, Das S, Kretzschmar W, Delaneau O, Wood AR, Teumer A, et al. A reference panel of 64,976 haplotypes for genotype imputation. Nat Genet. 2016;48:1279-1283.

3 Walter K, Min JL, Huang J, Crooks L, Memari Y, McCarthy S, et al. The UK10K project identifies rare variants in health and disease. Nature. 2015;526:82-90.

4 Dilthey A, Leslie S, Moutsianas L, Shen J, Cox C, Nelson MR, et al. Multi-population classical HLA type imputation. PLoS computational biology. 2013;9:e1002877.

5 Anderson CA, Pettersson FH, Clarke GM, Cardon LR, Morris AP, Zondervan KT. Data quality control in genetic case-control association studies. Nat Protoc. 2010;5:1564-1573.

6 Meng X, Li X, Timofeeva MN, He Y, Spiliopoulou A, Wei WQ, et al. Phenome-wide Mendelian-randomization study of genetically determined vitamin D on multiple health outcomes using the UK Biobank study. Int J Epidemiol. 2019;48:1425-1434.

7 Shim H, Chasman DI, Smith JD, Mora S, Ridker PM, Nickerson DA, et al. A multivariate genome-wide association analysis of 10 LDL subfractions, and their response to statin treatment, in 1868 Caucasians. PLoS One. 2015;10:e0120758.

8 Burgess S, Thompson SG, Collaboration CCG. Avoiding bias from weak instruments in Mendelian randomization studies. Int J Epidemiol. 2011;40:755-764.

9 Siggaard T, Reguant R, Jorgensen IF, Haue AD, Lademann M, Aguayo-Orozco A, et al. Disease trajectory browser for exploring temporal, population-wide disease progression patterns in 7.2 million Danish patients. Nat Commun. 2020;11:4952.

10 Schmidt M, Schmidt SA, Sandegaard JL, Ehrenstein V, Pedersen L, Sørensen HT. The Danish National Patient Registry: a review of content, data quality, and research potential. Clin Epidemiol. 2015;7:449-490.

11 Helweg-Larsen K. The Danish Register of Causes of Death. Scand J Public Health. 2011;39:26-29.

12 Schafmayer C, Harrison JW, Buch S, Lange C, Reichert MC, Hofer P, et al. Genome-wide association analysis of diverticular disease points towards neuromuscular, connective tissue and epithelial pathomechanisms. Gut. 2019;68:854-865.

13 Maguire LH, Handelman SK, Du X, Chen Y, Pers TH, Speliotes EK. Genome-wide association analyses identify 39 new susceptibility loci for diverticular disease. Nat Genet. 2018;50:1359-1365.

14 Law PJ, Timofeeva M, Fernandez-Rozadilla C, Broderick P, Studd J, Fernandez-Tajes J, et al. Association analyses identify 31 new risk loci for colorectal cancer susceptibility. Nat Commun. 2019;10:2154.

15 Burgess S, Scott RA, Timpson NJ, Davey Smith G, Thompson SG, Consortium E-I. Using published data in Mendelian randomization: a blueprint for efficient identification of causal risk factors. Eur J Epidemiol. 2015;30:543-552.

16 Bowden J, Davey Smith G, Burgess S. Mendelian randomization with invalid instruments: effect estimation and bias detection through Egger regression. Int J Epidemiol. 2015;44:512-525.
